# Finasteride delays atherosclerosis progression in mice and is associated with a reduction in plasma cholesterol in men

**DOI:** 10.1016/j.jlr.2024.100507

**Published:** 2024-01-23

**Authors:** Patrick McQueen, Donald Molina, Ivan Pinos, Samuel Krug, Anna J. Taylor, Michael R. LaFrano, Maureen A. Kane, Jaume Amengual

**Affiliations:** 1 Division of Nutritional Sciences, University of Illinois Urbana Champaign, Urbana, IL, USA; 2 Department of Food Science and Human Nutrition, University of Illinois Urbana Champaign, Urbana, IL, USA; 3 Department of Pharmaceutical Sciences, University of Maryland School of Pharmacy, Baltimore, MD, USA; 4 Carver Metabolomics Core, Roy J. Carver Biotechnology Center, University of Illinois Urbana Champaign, Urbana, IL, USA

**Keywords:** testosterone, dihydrotestosterone, hepatocyte, sex hormones, atheroma

## Abstract

Finasteride is commonly prescribed to treat benign prostate hyperplasia and male-pattern baldness in cis men and, more recently, trans individuals. However, the effect of finasteride on cardiovascular disease remains elusive. We evaluated the role of finasteride on atherosclerosis using low-density lipoprotein (LDL) receptor-deficient (*Ldlr*^*−/−*^) mice. Next, we examined the relevance to humans by analyzing the data deposited between 2009 and 2016 in the National Health and Nutrition Examination Survey. We show that finasteride reduces total plasma cholesterol and delays the development of atherosclerosis in *Ldlr*^*−/−*^ mice. Finasteride reduced monocytosis, monocyte recruitment to the lesion, macrophage lesion content, and necrotic core area, the latter of which is an indicator of plaque vulnerability in humans. RNA sequencing analysis revealed a downregulation of inflammatory pathways and an upregulation of bile acid metabolism, oxidative phosphorylation, and cholesterol pathways in the liver of mice taking finasteride. Men reporting the use of finasteride showed lower plasma levels of cholesterol and LDL-cholesterol than those not taking the drug. Our data unveil finasteride as a potential treatment to delay cardiovascular disease in people by improving the plasma lipid profile.

Atherosclerosis is the main underlying factor responsible for cardiovascular disease (CVD), the leading cause of death worldwide (1). Upon the entry of cholesterol-rich lipoproteins into the intima layer of the arterial wall, monocytes cross the arterial endothelium and differentiate into macrophages. The accumulation of lipoproteins, extracellular material, and lipid-laden macrophages in the arterial wall results in atherosclerotic lesions that can either occlude an artery or rupture to form a thrombus. These events can result in stroke, peripheral artery disease, and/or coronary heart disease (2).

Imbalance in sex hormones is a risk factor for CVD (3). While estrogen levels are generally considered cardioprotective in men and women, the role of androgens in cardiovascular health is not fully understood (4, 5, 6, 7). Testosterone is the primary androgen in men, and upon reaching target tissues, it exerts its function at the level of gene expression. Once in the cell, testosterone can be converted to its active metabolite dihydrotestosterone (5α–DHT) by the action of either the steroid 5α reductase type 1 (SRD5A1) or type 2 (SRD5A2). Several tissues express SRD5A1, while SRD5A2 is primarily present in the prostate and the hair follicle, where the formation of 5α–DHT can cause tissue hyperplasia and male pattern hair loss, respectively (8, 9).

In the 1980s, Merck & Co. developed the first SRD5A2 inhibitor, finasteride (10, 11). Finasteride is a front-line medication used to prevent and treat prostate hyperplasia. To date, it is the only US Food and Drug Administration (FDA) approved oral drug for treating androgenic alopecia in both cis male and trans individuals (FDA.gov). Recently, finasteride has been identified as a potential therapeutic target for opioid abuse, highlighting the pleiotropic effects of this commonly used medication (12). However, while finasteride is among the top 100 most prescribed drugs in the United States, its effects on atherosclerosis and CVD remain elusive (10, 13, 14).

The goal of this study was to examine the effect of finasteride on plasma cholesterol and atherosclerosis. To this end, we fed the low-density lipoprotein receptor (LDLR)-deficient (*Ldlr*^*−/−*^) mice (15) with increasing doses of finasteride. Using the National Health and Nutrition Examination Survey (NHANES) database, we also explored the relationship between finasteride intake and circulating lipid and glucose parameters, which are established risk factors for the development of CVD in humans (16, 17).

## Materials and Methods

### Animal studies

#### Diet composition and preparation

The dosage, administration route, and duration of finasteride interventions in rodents varies depending on the experimental approach, reaching a maximum dose of 4.5 mg of finasteride/day (18, 19, 20, 21). Because our primary outcome was to examine the effect of finasteride on atherosclerosis, we based the dose of finasteride on Liu's study with apoE-deficient mice, which used 1.5 mg finasteride/day/mouse (19). To this end, we fixed our maximum dose of finasteride at 1000 mg of finasteride/kg diet. Because mice typically eat 2.5 g of Western diet/day, this dosage corresponded to approximately 2.5 mg of finasteride/day/mouse (see supplemental Table S1 for average food intake results). Based on these calculations, we scaled down the content of finasteride in our diets to 100 and 10 mg of finasteride/kg diet to evaluate dose-response effects of the drug.

Finasteride was purchased from Cayman Chemical (Ann Arbor, MI) and shipped to Research Diets (New Brunswick, NJ) for diet preparation using cold extrusion to preserve finasteride's integrity. Upon arrival, diets were kept frozen at −20°C until use. To induce atherosclerosis, we fed four-week-old male mice a Western diet containing 0.3% cholesterol and 41% fat for 12 weeks, as done in the past (22, 23). Western diet with 0 mg/kg of finasteride was prepared and stored under the same conditions.

#### Mouse husbandry

We used male *Ldlr*^*−/−*^ (#002207, The Jackson Laboratory, Bar Harbor, ME) for all our experiments. All mice were housed and nurtured at 24°C on a 12-h light/dark cycle and fed ad libitum with free access to water. The dams and pups were fed a nonpurified standard breeder diet (Teklad global 18% protein diet: Envigo, Indianapolis, IN). Pups were weaned three weeks after birth onto a breeder diet for an additional week, and then fed Western diet without finasteride or the same diet supplemented with 10, 100, and 1000 mg finasteride/kg of diet for 12 weeks.

Because finasteride is typically prescribed to cis men, all the experiments were performed using male mice. Mice born from the same litter were ear-tagged and weaned into separate cages in the company of mice born from other litters with a maximum of three days apart. All cages were monitored daily during the first week and then weekly to detect signs of stress or aggression.

Body weights and food intake were measured once a week during the entire experiment. After 12 weeks on a diet, mice were fasted overnight and euthanized by injecting intraperitoneally a lethal dose of 80 mg ketamine/kg and 8 mg/kg of xylazine. Blood was drawn from the heart's right ventricle using EDTA-coated syringes and kept on ice for plasma preparation. Mice were perfused with 10% sucrose in saline solution (0.9% NaCl in water) for approximately two minutes. Upon harvesting, tissues were immediately placed in liquid nitrogen and stored at −80°C. Aortic roots were cleaned of surrounding fat and tissue under a microscope, embedded in optimal cutting temperature compound (Sakura, Torrance, CA), placed on dry ice, and stored at −80°C. Plasma was collected by centrifugation of the blood for five minutes at 1000 *g* and stored immediately at −80°C.

#### Analysis of liver toxicity

Circulating alanine transaminase and aspartate transaminase activities were analyzed using commercially available kits (Abcam, Cambridge, MA), following the manufacturer's instructions. Plasma microRNA-122 (miR-122), a highly sensitive indicator of liver toxicity (24), was analyzed as previously described (25). Briefly, total plasma RNA was isolated and retrotranscribed to complementary DNA. Samples were spiked with synthetic *Caenorhabditis elegans* miR-39 (cel-miR-39) as external control, and quantitative real-time PCRs were performed using TaqMan Fast Advanced Master Mix (Applied Biosystems). Expression levels were calculated using the Pfaffl method.

#### Hepatic and plasma lipid analyses

Total lipid levels in the liver were quantified following the Folch method (26). Plasma total cholesterol, cholesterol found in the HDL fraction (HDL-C), and triglyceride concentrations were measured using colorimetric assays (FUJIFILM Wako Diagnostics, Mountain View, CA) and performed in accordance with the manufacturer's instructions.

#### Bile acid measurements

Bile acid measurements were performed using liquid chromatography-tandem mass spectrometry (LC-MS/MS) as previously described (27, 28, 29). Briefly, 100 μl of plasma were loaded directly onto the ISOLUTE PLD+ column per manufacturer’s instructions, and 5 μl of internal standard (1 μg/ml deuterated bile acids stock) was added to each tube. Samples were extracted with 400 μl of acetonitrile, vortexed for 30 s, and then eluted through the column slowly with no more ant 4 psi applied via positive pressure manifold. Eluent was dried down under nitrogen at 40°C and reconstituted with 100 μl of water:acetonitrile (50:50).

LC-MS/MS analysis was performed according to previously published methodology on a Waters I-Class ultra performance liquid chromatography coupled to a Waters TQ-XS tandem quadrupole mass spectrometer with LC-MS/MS conditions summarized in supplemental Table S2 (28). Expected retention time and LC-MS/MS acquisition parameters for each individual analyte in the three timed multiple reaction monitoring (MRM) segments are given in supplemental Table S3. Abbreviations for individual bile acids are listed in the supplementary section.

For liver samples, we first weighed tissues and homogenized them using Precellys and 20x the volume of ice cold acetonitrile:water (1:1) (i.e. 50 mgs of tissue = 1000 μl of solvent added). Homogenizer was set to 5500 rpm for 30 s interval and pulsed twice. Homogenate was centrifuged at 4000 rpm for 5 min, and 600 μl of supernatant was combined with 30 μl of internal standard and 3 ml of 5% ammonium hydroxide to precipitate proteins. Samples were then allowed to shake at room temperature for 2 h and the organic layer was then passed through the PLD+ column and reconstituted as listed above (27, 28, 29).

Total bile acids is a sum of all quantifiable bile acids; unconjugated bile acids is a sum of CA, LCA, DCA, CDCA, and UDCA; glycine-conjugated bile acids is a sum of GCA, GCDCA, GLCA, and GUDCA; taurine -conjugated bile acids is a sum of TCA, TCDCA, TLCA, and TUDCA.

#### Steroid measurements

Samples were analyzed for steroids using LC-MS/MS by the Carver Metabolomics Core of the Roy J. Carver Biotechnology Center, University of Illinois Urbana Champaign. Steroids chemical standards used to create calibration curves for quantification were acquired from Cayman chemical (Ann Arbor, MI, USA) and Sigma-Aldrich (St. Louis, MO, USA). At the beginning of the extraction process, 20 μl of 1 μg/ml of labeled surrogate internal standards (testosterone-d3, estradiol-d4, and progesterone-d9) were spiked into each liver sample. Plasma samples were spiked with 4 μl of 10 μg/ml of the same labeled surrogate internal standards mixture. The liver tissue (115–150 mg) underwent bead beating in 500 μl methanol, while plasma (25 μl) was vortexed with 75 μl methanol. Upon centrifugation, supernatants were transferred to HPLC vials and subsequently analyzed. Chromatography was performed on an Agilent 1290 Infinity II UHPLC system (Agilent, Santa Clara, CA, USA), with a Waters Acquity C18 BEH 2.1 × 150 mm, 1.7 μm; Column Temp −60°C; flow rate 450 μl/min. Mobile phases consisted of 0.1% formic acid in water (A) and 0.1% formic acid in acetonitrile (B). Data were collected on a Sciex 6500+ triple quadrupole mass spectrometer (Sciex, Framingham, MA, USA). Data were acquired in positive ionization mode. MRM was used for detection. Peak integration and quantitation using calibration curves adjusted for internal standards were performed using Sciex OS MultiQuant 3.1 software (https://sciex.com/products/software/sciex-os-software). Expected retention time and LC-MS/MS acquisition parameters for each individual analyte in the three timed MRM segments are given in supplemental Table S4.

#### Triglyceride secretion rate

After overnight fasting*, Ldlr*^*−/−*^ mice fed Western diet without finasteride or the same diet containing 1000 mg/kg of finasteride for 12 weeks were injected with 1,000 mg/kg pluronic F127 poloxamer-407 (Sigma-Aldrich) to inhibit lipoprotein clearance from plasma (22, 30). Blood samples were collected every hour from the tail for four hours to determine the triglyceride secretion rate using commercially available kits (FUJIFILM Wako Diagnostics). As done in the past (31), we represented secretion rate as the average of the slope referred to the mice fed Western diet without finasteride.

#### Cholesterol absorption dual-isotope method

After overnight fasting*, Ldlr*^*−/−*^ mice fed Western diet without finasteride or the same diet containing 1000 mg/kg of finasteride for 12 weeks were gavaged 200 μl olive oil containing 1 μCi [14C]cholesterol (American Radiolabeled Chemicals, St. Louis, MO) and 2 μCi [3H]sitostanol (American Radiolabeled Chemicals), as done in the past (22). We estimated cholesterol uptake by collecting the feces every 24 h during four consecutive days.

We dried the feces using a SpeedVacuum concentrator (Eppendorf, Hamburg, Germany) and ground them into powder. Approximately 100 mg feces per sample were used and dissolved overnight in water and saponified with potassium hydroxide for 2 h. The total lipid content was extracted twice with a mixture of diethylether:hexane:ethanol (66:33:1). The supernatants of all samples were collected and evaporated in a SpeedVac and resuspended in scintillation liquid cocktail (PerkinElmer). [14C]cholesterol and [3H]sitostanol were measured with an LS6500 scintillation counter (Beckman, Brea, CA). The calculation of the percentage of cholesterol absorption followed Wang and Carey’s methodology (32).

#### Fast-performance liquid chromatography (FPLC) analyses

Plasma lipoproteins were fractionated using FPLC with two Superose 6 10/300 GL columns (GE HealthCare, Boston, MA) on a Shimadzu HPLC system (Columbia, MD) as done in the past (22, 33). Pooled samples (five mice/group) were used for analysis.

Lipoprotein fractions were identified by measuring lipid content (cholesterol and triglyceride) and Western blot analysis for apolipoprotein B (apoB) and apolipoprotein A-I (apoA-I).

#### Western blot analyses

Five to ten μl for each FPLC fraction were separated by sodium dodecyl-sulfate polyacrylamide gel electrophoresis (SDS-PAGE), transferred to nitrocellulose membrane (Bio-Rad, Hercules, CA), and incubated with either rabbit apoB (Millipore-Sigma, Burlington, MA) or goat apoA-I (Meridian Life Science Inc, Memphis, TN) primary antibodies. HRP-conjugated secondary antibodies were prepared in Tris-buffered saline (15 mM NaCl and 10 mM Tris/HCl, pH 7.5) containing 0.01% Tween 20 with 5% fat-free milk powder and incubated for 1 h at room temperature. Immunoblots were developed with the enhanced chemiluminescence system (Thermo Fisher Scientific, Waltham, MA) and scanned.

#### *En face* plaque area quantification in the aortic arch

The plaque area in the aortic arch was estimated by quantifying lesion size present in the ascending and upper section of the descending aorta, the brachiocephalic artery, the left carotid artery, and the left subclavian artery. We also represent the lesion size in the brachiocephalic artery which develops advanced lesions in mice (34). Images were taken using a CX3-2300S-JW11 Zoom Stereo Microscope (Omano, China) and analyzed with ImageJ software (NIH; https://imagej.net/ij/). Lesion size was estimated to the lesion area occupied and represented as a percentage.

#### Aortic root analyses

Aortic roots were sectioned using a cryostat (Leica CM3050 S, Leica Biosystems, Buffalo Grove, IL) at a thickness of 6 μm. The sections were stained with anti-CD68 (clone FA-11; Bio-Rad) to quantify macrophage content in the plaque and counterstained with hematoxylin/eosin to visualize the structure of the lesion, as previously done (22). Necrotic core area quantification was performed as done in the past (23). Images were taken using a Zeiss Axioskop 40 light microscope (Carl Zeiss, Stuttgart, Germany) at 10x magnification, and all quantifications were performed using the ImageJ software (NIH).

#### Flow cytometry and monocyte recruitment

Circulating monocytes were labeled with 5-ethynyl-2′-deoxyuridine (EdU) to examine monocyte recruitment into atherosclerotic lesions, as described previously (35). Mice were injected intraperitoneally with 250 μl containing 1 μM EdU in saline (Invitrogen, Waltham, MA) two days before the sacrifice. To measure monocyte labeling efficiency, we examined EdU+ monocytes in circulation 48 h after injection by flow cytometry. We incubated blood samples with red blood cell lysis buffer (BioLegend, San Diego, CA) and stained them with primary antibodies antimouse CD45-FITC (Clone 30-F11; BioLegend), CD115-APC (Clone AFS98; BioLegend), and Ly6c-PerCP/Cy5.5 (Clone RB6-8C5; BioLegend) to identify circulating monocytes. Cells were permeabilized, and EdU was detected using Click-IT EdU Pacific Blue Flow Cytometry Assay Kit (Invitrogen). Flow cytometry was performed in LSR II analyzer (B.D. Biosciences, Franklin Lakes, NJ), and data were analyzed using FCS Express 5.01.0082 (De Novo Software, Pasadena, CA; https://denovosoftware.com/).

#### Immunostaining

Aortic root sections were fixed in 10% formalin, permeabilized with 1% Triton X-100 in phosphate buffer, and stained for EdU using Click-IT EdU Imaging Kit with Alexa Fluor 647 azide (Invitrogen) to visualize EdU+ cells in the lesions. Macrophage proliferation was determined by staining slides with the proliferative marker Ki67, using an anti-Ki67 antibody (clone SP6; Abcam). Newly recruited monocytes in lesions were identified as CD68+EdU+Ki67-cells. CD68+EdU-Ki67+ cells were considered proliferating macrophages. Images were taken using a Zeiss Axioscan.Z1 slide scanner (Carl Zeiss), and quantifications were performed using the ImageJ software (NIH).

Liver sections were fixed with 10% formalin, permeabilized with 0.5% Triton X-100, and stained with the macrophage marker F4/80 (clone A3-1; Abcam). F4/80+ cells were visualized using a secondary antibody conjugated to Alexa 555 (goat anti-rat, Thermo Fisher Scientific) and counterstained with 4',6-diamidino-2-phenylindole.

#### RNA sequencing and bioinformatic analyses

Liver RNA was isolated using a Zymo Research mini prep tissue kit (Irvine, CA), following a DNaseI treatment, according to the manufacturer's instructions. Samples were sequenced using the HiSeq 4000 Paired-End (Illumina, San Diego, CA), and the results were analyzed using Gene Set Enrichment Analysis (GSEA) software v4.2.2 (UC San Diego, CA; Broad Institute, MA; https://www.gsea-msigdb.org/gsea/index.jsp). The data discussed in this publication have been deposited in NCBI's Gene Expression Omnibus and are accessible through the GEO Series accession number GSE255708 (https://www.ncbi.nlm.nih.gov/geo/query/acc.cgi?acc=GSE255708).

#### Glucose tolerance test

A week before tissue harvesting, we fasted the mice overnight in clean cages with free access to water. In the morning, mice were gavaged with a saline solution containing 1.5 g of glucose/kg of body weight. Blood glucose levels were measured with a glucometer (OneTouch) using blood from tail snips, as described previously (36).

#### Statistical analyses

Data are expressed as means ± SEM. All statistical differences were analyzed using GraphPad Prism software (San Diego, CA). Data normality were analyzed using Shapiro–Wilk normality tests. Data comparing all four experimental groups were analyzed either using two-way ANOVA with Dunnett's multiple comparison testing or repeated-measures two-way ANOVA with Dunnett's multiple comparisons test. Data comparing the finasteride-free Western diet group to high-dose finasteride were analyzed using a two-tailed student's *t* test. For all cases, statistical significance was set at *P* < 0.05. Data extracted from the RNA sequencing results were represented as means ± SEM, but statistical significance was set at a false discovery rate (FDR) <0.05.

### Retrospective human study

The NHANES is an ongoing, cross-sectional database that surveiles the health and nutrition status of the noninstitutionalized United States population. NHANES is administered through the National Center for Health Statistics, a government agency within the Centers for Disease Control and Prevention. A comprehensive report on the methods of NHANES data collection can be found in previous publications (37). The study was conducted according to the guidelines set forth in the Declaration of Helsinki.

#### Study sample and inclusion criteria

Data collected between 2009 and 2016 were extracted from the US NHANES database. Because finasteride is typically taken to treat male baldness and benign prostatic hyperplasia, women were excluded from the analyses. Only those men who completed the 24-h health and dietary recall interviews and participated in the Mobile Examination Center sample collection were included in the study. Further, subjects with prostate inflammation and infection, diagnosed or self-reported with prostate cancer, were excluded from the study. While running our initial models, we observed an imbalance in individuals taking finasteride, where only 10 out of 165 were <50 years of age. Given this imbalance, we included only those 50 or older.

#### Variables and measurements

The dependent variables for the study were total cholesterol, cholesterol found in the LDL fraction (LDL-C), HDL-C, triglyceride, glucose, and glycohemoglobin in plasma. Subjects were categorized as finasteride users or not. The following variables were considered as covariates: alcohol intake (derived from ALQ150 and ALQ151); confirmed diagnosis of coronary heart disease (derived from MCQ160C); any kind of liver condition (derived from MCQ160L); cancer or malignancy of any kind (derived from MCQ220); diagnosis with diabetes or prediabetes, and insulin or other diabetic medication users (derived from DIQ050 and DIQ070; DIQ010 and DIQ160); diagnosis with high blood cholesterol level and cholesterol medication users (derived from BPQ080 and BPQ090D); smoking status (derived from SMQ040); drugs affecting lipid profile; drugs affecting glucose profile; age; body mass index (categories derived from BMXBMI); and race (RIDRETH1).

#### Statistical analyses

Statistical analyses were performed using SAS v9.4 software (SAS Institute, Cary, NC; https://www.sas.com/en_us/software/viya.html?utm_source=other&utm_medium=cpm&utm_campaign=non-cbo-us&dclid=&gclid=CjwKCAiAlJKuBhAdEiwAnZb7lSuxrO759Q9xT8bOqgn3boaawkIKcEQ71GtIZG-Q102ryDBmv5natRoCGqwQAvD_BwE). Descriptive statistics were calculated using PROC MEANS and PROC FREQ procedures to examine the association between finasteride intake and the outcome variables of total cholesterol, LDL-C, HDL-C, triglyceride, glucose, and glycohemoglobin. Bivariate analyses for each outcome and finasteride intake were conducted separately with each of the covariates (listed in previous section) to assess (a) if these variables truly were confounding the relationship between finasteride intake and outcome variable (change of >10% in beta coefficient) and (b) if these variables qualified as interaction terms with finasteride use in the model for each of the six outcomes (at *P* < 0.15).

A subset of candidate confounders and interacting variables were identified for each outcome model (data not shown). Multiple linear regression was conducted for each of the six outcomes where selected variables were controlled as confounders and considered for interaction effects. PROC SURVEYREG procedure was used in these analyses, and least square means estimates were assessed for each significant interaction (at *P* < 0.15) in the final models. Each model was built for patients over 50 years of age since less than ten patients between 19 and 50 years of age took finasteride. All analyses were conducted according to the NHANES analytical guidelines. The masked variance was accounted for, and appropriate sample weights were applied to represent unequal probabilities of selection, nonresponse bias, and oversampling. Eight-year weights were calculated using the method provided by National Center for Health Statistics.

#### Study approval

Animal studies were executed in compliance with the guidelines published in the National Institutes of Health's Guide for the Care and Use of Laboratory Animals (38). The animal protocols were approved by the Institutional Animal Care and Use Committee of the University of Illinois at Urbana Champaign. All participants of the NHANES must provide written consent for the public availability and use of these data. This process is completed under the approval of the National Center for Health Statistics Institutional Review Board.

## Results

### Mouse study–high dose of finasteride decreases plasma cholesterol and triglyceride levels in *Ldlr*^*−/−*^ mice

**Fig. 1:**
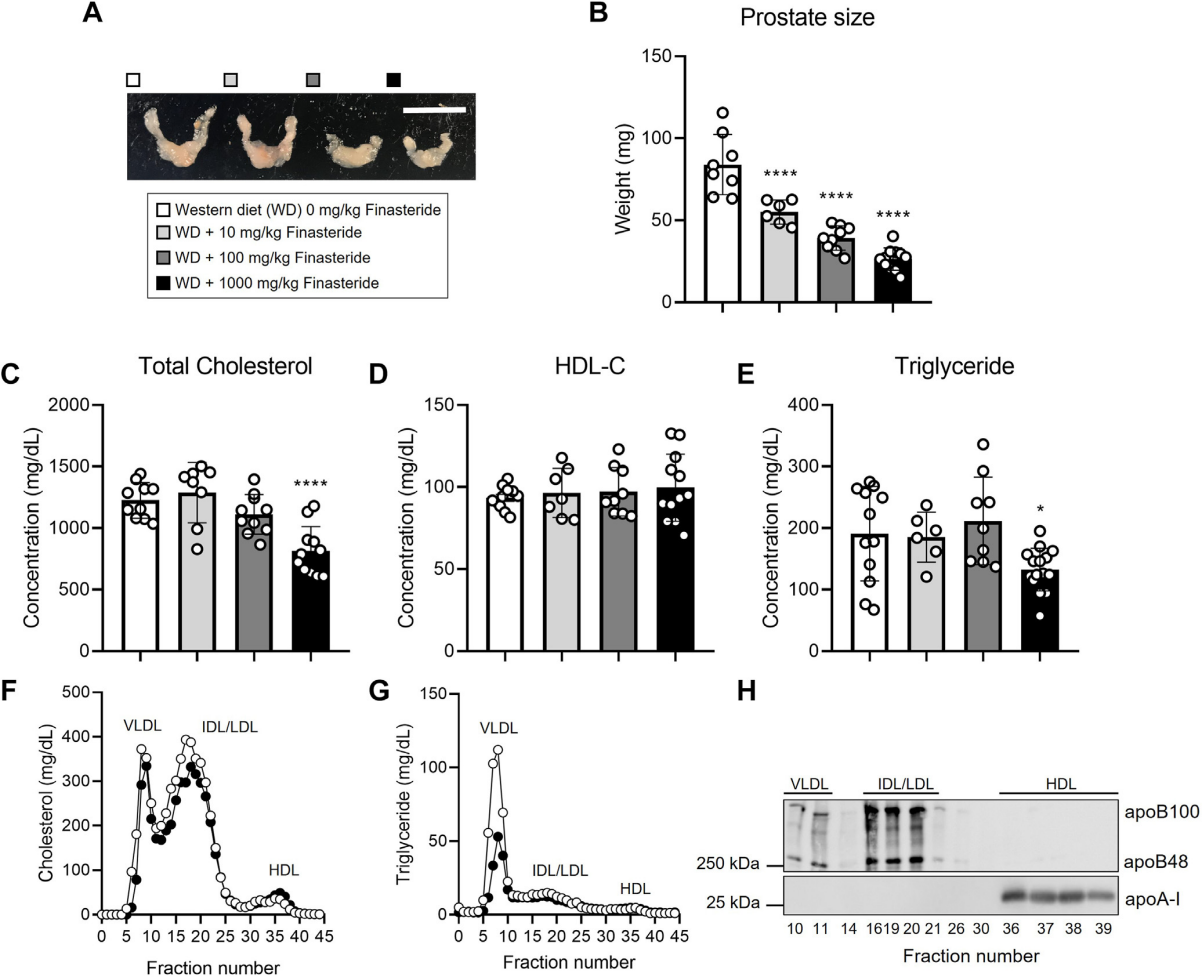
Finasteride supplementation reduces total cholesterol and triglyceride plasma levels in *Ldlr*^*−/−*^ mice. Four-week-old male *Ldlr*^*−/−*^ mice were fed Western diet with increasing doses of finasteride for 12 weeks before tissue and blood harvesting. (A and B) prostate size at the moment of the sacrifice. (C) total cholesterol, (D) cholesterol levels in the HDL fraction (HDL-C), and (E) triglyceride plasma levels. (F) cholesterol and (G) triglyceride distribution in FPLC-fractioned plasma (data pooled from 5 mice/group) fed Western diet without finasteride or the same diet containing 1000 mg/kg of finasteride. (H) representative Western blot analysis of plasma apoB and apoA-I in selected FPLC fractions. Data represent the mean ± SEM. Statistical differences were evaluated using one-way ANOVA (*P* < 0.05). n = 6 to 15 mice/group. ∗*P* < 0.05, ∗∗∗∗*P* < 0.001 considering *Ldlr*^*−/−*^ mice fed Western diet without finasteride as reference group. Size bar = 1 cm. apoB, apolipoprotein B; apoA-I, apolipoprotein A-I; FPLC, fast-performance liquid chromatography; HDL, high-density lipoprotein; HDL-C, cholesterol found in the HDL fraction; LDLR, low-density lipoprotein receptor.

To determine the implication of finasteride in the development of atherosclerosis, we examined the role of this compound in the atheroprone *Ldlr*^*−/−*^ mouse model (15). We fed male *Ldlr*^*−/−*^ mice a Western diet containing increasing doses of finasteride (10, 100, and 1000 mg/kg of diet) for 12 weeks or the same diet without finasteride (0 mg/kg). Final body weights remained unaltered between the groups, as well as the food intake and adiposity, which represents the sum of the inguinal, gonadal, and retroperitoneal white adipose tissue divided by total body weight (supplemental Table S1). Mice fed finasteride at the dose of 1000 mg/kg of diet showed a reduction in the liver size compared to mice fed diet without finasteride (supplemental Table S1). Finasteride did not affect classical markers of liver damage, such as circulating transaminase activities, nor the levels of miRNA-122, a novel marker of liver toxicity (24, 39) (supplemental Table S1). Finasteride reduced prostate size in a dose-dependent manner, in agreement with previous findings in rodents and humans (40, 41) (Fig. 1A, B).

We next examined the effect of finasteride on plasma lipid levels. Only those mice fed a Western diet containing 1000 mg/kg of finasteride experienced a reduction in total circulating cholesterol without affecting HDL-C levels compared to the mice fed without finasteride (Fig. 1C, D). Mice fed 1000 mg/kg of finasteride also showed a reduction in circulating triglycerides compared to those fed a finasteride-free diet (Fig. 1E). Using pooled samples, we compared plasma cholesterol and triglyceride partitioning between finasteride-free and 1000 mg/kg finasteride-fed mice by FPLC. Very low-density lipoprotein (VLDL) and LDL fractions in finasteride-fed mice showed a reduction in total cholesterol and triglyceride content in comparison to mice fed a diet without finasteride (Fig. 1F–H).

### Mouse study—finasteride supplementation reduces atherosclerotic lesion size in *Ldlr*^*−/−*^ mice

**Fig. 2:**
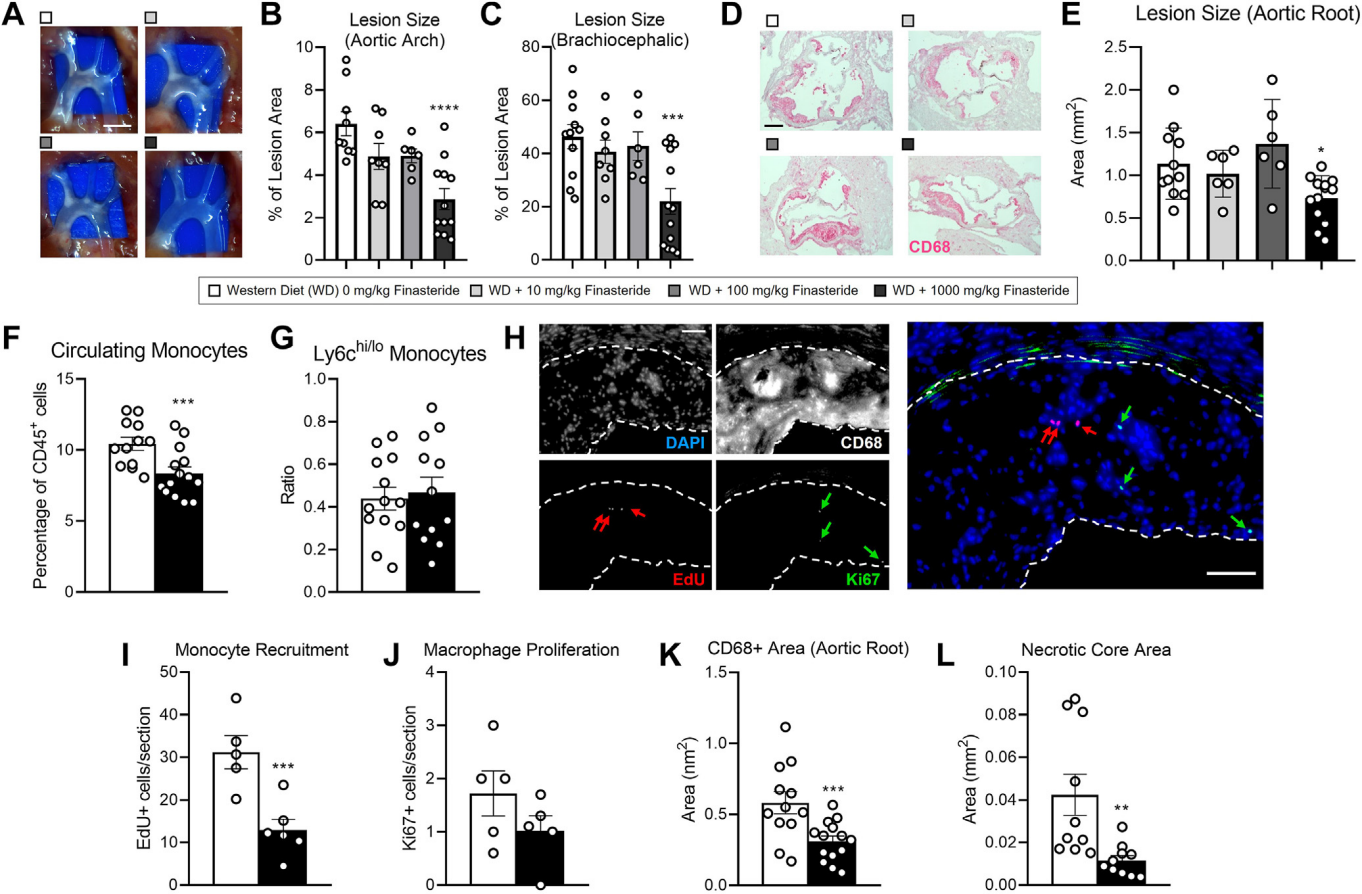
Finasteride decreases plaque size and macrophage content in *Ldlr*^*−/−*^ mice. Four-week-old male *Ldlr*^*−/−*^ mice were fed Western diet with increasing doses of finasteride for 12 weeks. Two days before tissue harvesting, mice were injected with EdU to track monocyte trafficking into the atherosclerotic lesions. (A) *En face* visualization of atherosclerotic lesions in the aortic arch, (B) and quantification of relative plaque content at the level of the entire aortic arch, and (C) the brachiocephalic artery. (D) histological sections at the level of the aortic root stained for CD68 (magenta) and counterstained with hematoxylin. (E) lesion area quantification. (F) circulating monocytes, (G) and ratio between monocytes expressing high and low levels of the surface marker Ly6c (Ly6c^hi/lo^) measured by flow cytometry at the moment of the sacrifice. (H) histological sections stained for EdU, Ki67, and CD68. DAPI was used to stain nuclei. Atherosclerotic lesion is outlined in dotted white line. (I) quantification of newly recruited monocytes, and (J) proliferative CD68+ cells in the lesion. (K) total CD68+ area, and (L) necrotic core area at the level of the aorta. Data represent the mean ± SEM. Statistical differences were evaluated using one-way ANOVA or two-tailed student's *t* test (*P* < 0.05). n = 6 to 15 mice/group. ∗*P* < 0.05, ∗∗*P* < 0.01, ∗∗∗*P* < 0.005, ∗∗∗∗*P* < 0.001 considering *Ldlr*^*−/−*^ mice fed Western diet without finasteride as reference group. Size bar = 2 mm (A) 200 μm (D and H). DAPI, 4',6-diamidino-2-phenylindole; LDLR, low-density lipoprotein receptor.

We next evaluated whether finasteride alters the formation of atherosclerotic lesions at the level of the aortic arch (Fig. 2A). *En face* lesion quantifications showed a reduction in the total plaque content in the aortic arch and the brachiocephalic artery only in those mice fed 1000 mg/kg of finasteride (Fig. 2B, C). These results agreed with those observed at the level of the aortic root (Fig. 2D, E).

Proinflammatory monocytes contribute to plaque development and growth by infiltrating the arterial wall, where they become macrophages (42). To examine the effect of finasteride on monocyte number and inflammatory status, we quantified the relative number of monocytes in the blood and examined the expression of the surface marker Ly6C by flow cytometry, respectively (42). Mice fed 1000 mg finasteride/kg showed a reduction in monocyte number in comparison to mice fed a diet without finasteride, although these changes were not accompanied by alterations in Ly6C expression (Fig. 2F, G). Next, we quantified the number of newly recruited monocytes, as well as macrophage proliferation by quantifying the number of EdU+ and Ki67+ cells, respectively (see methods for details) (Fig. 2H). Our results show that mice fed 1000 mg/kg of finasteride showed reduced monocyte recruitment without altering macrophage proliferation compared to mice fed a finasteride-free diet (Fig. 2I, J). Accordingly, macrophage content (CD68+ cells) in the plaque was reduced in finasteride-fed mice compared to those fed without finasteride (Fig. 2K).

Lastly, we evaluated the necrotic area in the lesions, a surrogate indicator of plaque stability in people (43). Mice fed 1000 mg/kg of finasteride presented a reduction in the necrotic core area compared to mice fed a diet without finasteride (Fig. 2L).

### Mouse study—effect of finasteride on hepatic transcriptional regulation and sex hormone levels in *Ldlr*^*−/−*^ mice

**Fig. 3:**
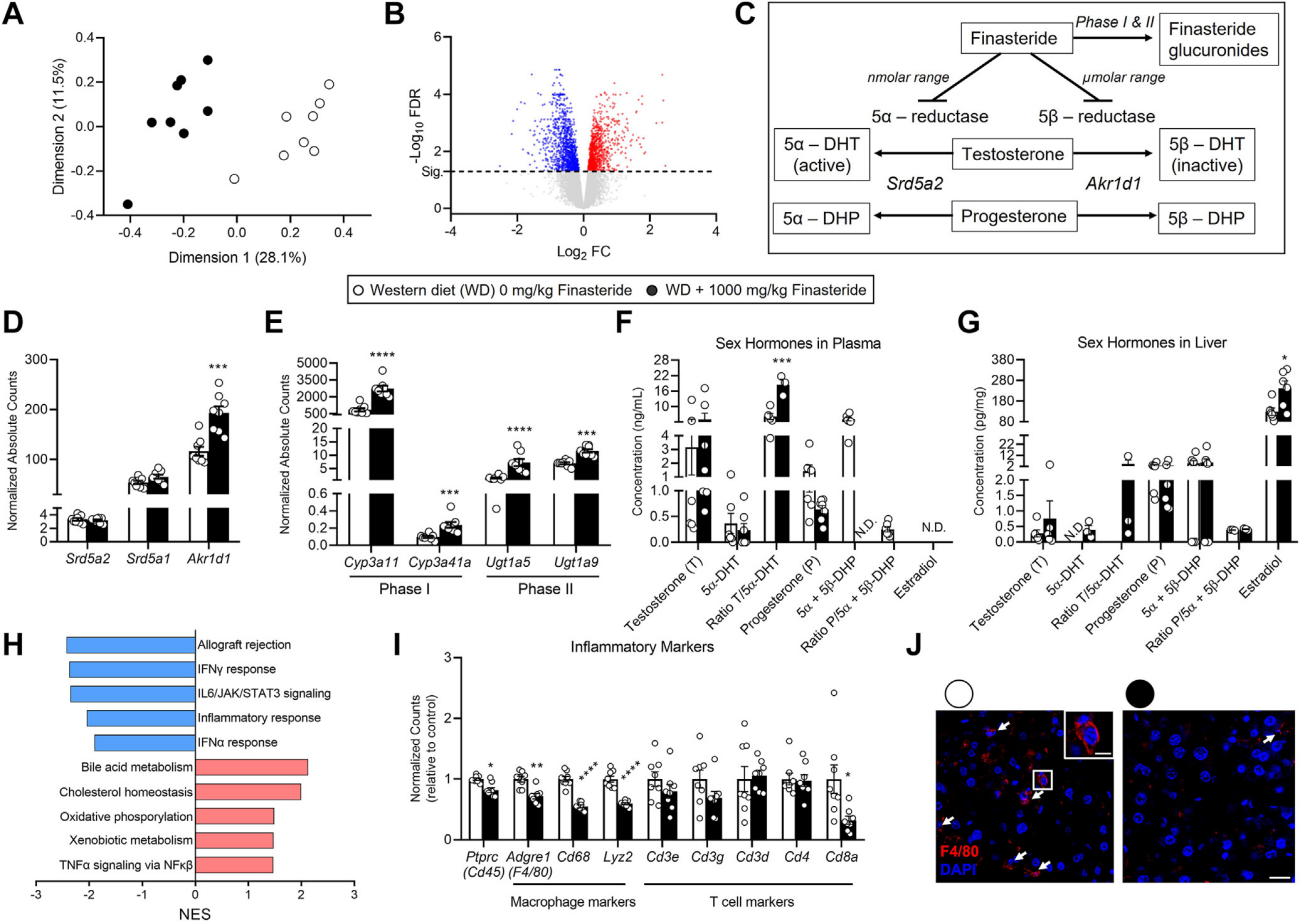
Finasteride alters hepatic gene expression and sex hormone levels in mice. Four-week-old male *Ldlr*^*−/−*^ mice were fed either Western diet with 1000 mg finasteride/kg or the same diet without finasteride for 12 weeks. Total liver mRNA was extracted and sequenced. (A) Principal component analysis, and (B) volcano plot representing genes significantly upregulated (red) and downregulated (blue) in response to finasteride supplementation. (C) Scheme highlighting the main targets of finasteride and it inhibits the enzymatic activity of SRD5A2 and AKR1D1, which are implicated in the desaturation of testosterone to dihydrotestosterone (DHT). (D) Effect of finasteride in the expression of the three enzymes implicated in DHT formation, (E) and elimination of finasteride. (F) Sex hormone levels in plasma and (G) and liver. (H) Top five up-regulated and downregulated GSEA pathways in response to finasteride exposure. (I) Expression of the lymphocyte marker (*Ptprc*), macrophage and T cell markers in the liver. (J) Liver sections showing F4/80 (macrophage marker) expression. Data represent the mean ± SEM. n = 6 to 10 mice/group. Data extracted from the RNA sequencing were evaluated using FDR. ∗ q < 0.05, ∗∗∗ q < 0.005, ∗∗∗∗ q < 0.001 considering *Ldlr*^*−/−*^ mice fed Western diet without finasteride as reference group. DHT; dihydrotestosterone. DHP; dihydroprogesterone. Size bar = 5 μm (magnified image) and 20 μm. AKR1D1, aldo-keto reductase family 1 member D1; FDR, false discovery rate; GSEA, Gene Set Enrichment Analysis; LDLR, low-density lipoprotein receptor; SRD5A, steroid 5α reductase.

*Ldlr*^*−/−*^ mice fed 1000 mg/kg of finasteride showed a reduction in plasma cholesterol in comparison to those fed with 0 mg/kg of finasteride (Fig. 1C). Importantly, plasma cholesterol values correlated with the lesion size in the aortic arch and root, suggesting that plasma cholesterol drives atherogenesis in our experimental model (supplemental Fig. S1A, B). Because the liver contributes to both systemic lipid metabolism and finasteride detoxification (44, 45), we performed transcriptomic profiling by RNA sequencing in the liver of *Ldlr*^*−/−*^ mice exposed to 1000 mg/kg of finasteride and their littermate finasteride-free diet-fed mice (Fig. 3A). A total of 2,102 genes were regulated with an FDR < 0.05 from which 1,009 were upregulated (Fig. 3B).

First, we examined the mRNA levels of proteins implicated in the conversion of testosterone to DHT and the degradation pathway of finasteride (Fig. 3C). Finasteride did not alter the expression of SRD5A1 or SRD5A2, both of which activate testosterone to form 5α-DHT. However, finasteride upregulated the expression of the aldo-keto reductase family 1 member D1 (AKR1D1). AKR1D1 participates in several metabolic pathways, including bile acid formation and the inactivation of steroid hormones such as testosterone and progesterone (46). The activity of AKR1D1 is also inhibited by finasteride. However, greater doses of the drug are necessary to inhibit the enzymatic action of AKR1D1 compared to SRD5A2, which is the primary target of finasteride (47) (Fig. 3C, D). Finasteride also upregulated the expression of *Cyp3a11* and *Cyp3a41a*, the murine orthologs for *CYP3A4* (48) that in humans is responsible for phase I metabolism of finasteride (44). The metabolites derived from finasteride during phase I are conjugated by the action of UDP-glucuronosyltransferase 1 (UGT1), which in rodents and humans are encoded by the *UGT1* gene to produce multiple UGT1A proteins (44, 49). As it occurred for the *CYP3A4* orthologs, finasteride upregulated the mRNA expression of two UGT isoforms; the *Ugt1a5* and *Ugt1a9* (Fig. 3E).

As previously mentioned, finasteride results in androgen imbalance through changes in steroid metabolism (50). Hence, we quantified the levels of the main sex hormones serving as upstream and downstream metabolites of SRD5A2 and AKR1D1 in plasma and the liver samples. In plasma, finasteride did not alter absolute levels of testosterone and 5α-DHT, but increased the testosterone/5α-DHT ratio. This metric is commonly utilized to estimate SRD5A2 activity in people, where high ratio values is an indicator of low SRD5A2 activity (51). Finasteride failed to alter progesterone levels in plasma. Dihydroprogesterone levels were detected in the mice fed without finasteride, but not in any of the finasteride-fed mice, further supporting that finasteride inhibited SRD5A2, and possibly AKR1D1 activities (52, 53) (Fig. 3C, F). We did not observe differences in hepatic testosterone, 5α-DHT, progesterone, or dihydroprogesterone levels, nor their respective ratios. However, we observed an increase in hepatic estradiol concentration in mice fed finasteride that may be indicative of elevated aromatase activity (Fig. 3G) (54).

Next, we performed a GSEA to examine which pathways were affected by finasteride in the liver. The top five downregulated pathways in response to finasteride suggested that this drug decreases inflammation in finasteride-fed mice compared to those fed without finasteride (Fig. 3H). These changes were accompanied by an overall downregulation of classical macrophage markers such as F4/80 (Fig. 3I). These changes were supported by immunostaining for F4/80 in liver sections, which resulted in a reduction in the number of F4/80+ cells in mice fed finasteride (Fig. 3J). While most T-cell markers remained unaltered, *Cd8a* was downregulated in response to finasteride, suggesting that finasteride could decrease the cellular content of macrophages and CD8+ cells in the liver (Fig. 3I).

### Mouse study—effect of finasteride on hepatic lipid metabolism

**Fig. 4:**
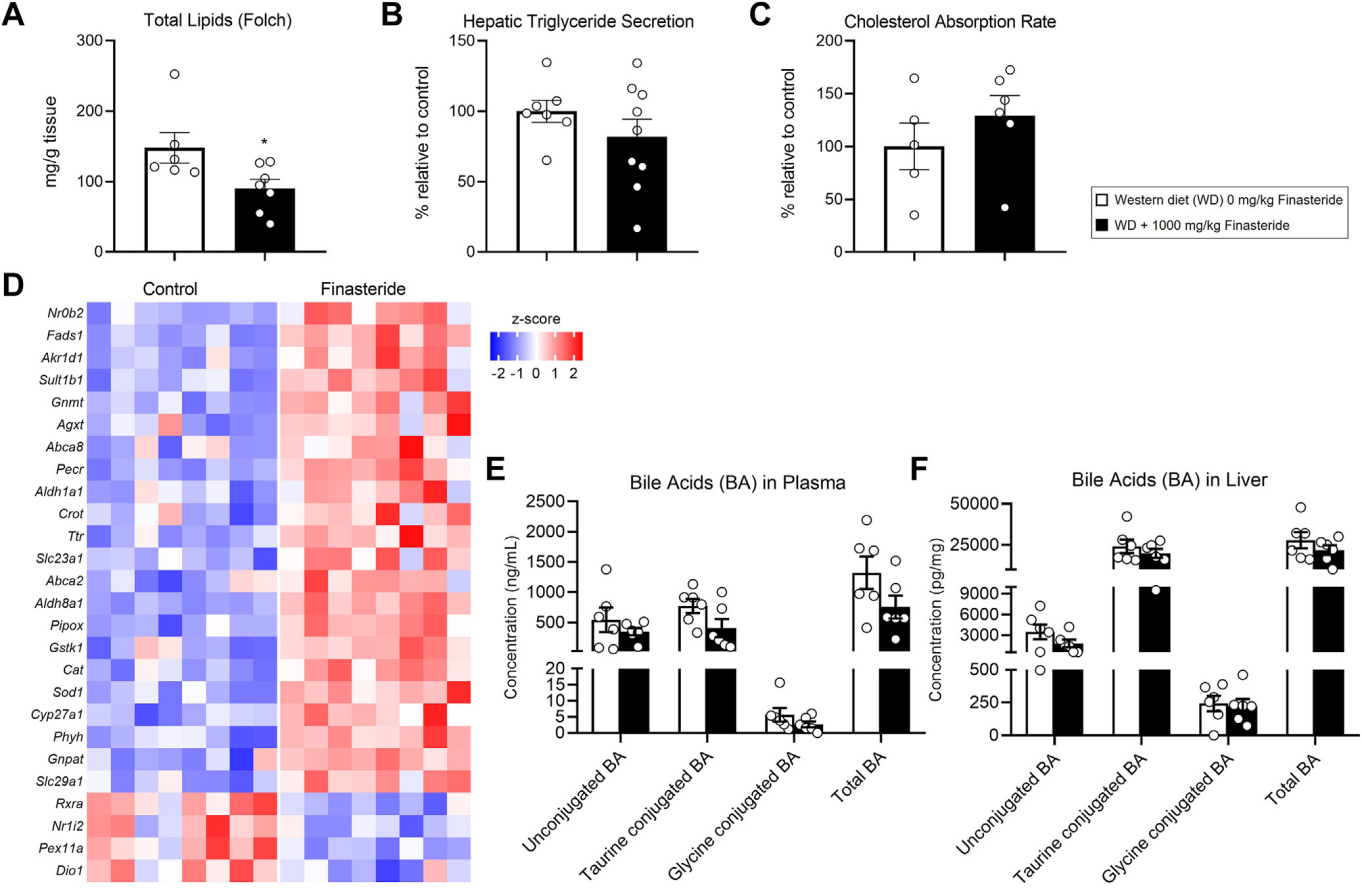
Effect of finasteride on lipid and bile acid metabolism in mice. Four-week-old male *Ldlr*^*−/−*^ mice were fed either Western diet with 1000 mg finasteride/kg or the same diet without finasteride for 12 weeks. Total liver mRNA was extracted and sequenced. (A) Total lipid content in liver homogenates, (B) relative triglyceride secretion rate, and (C) intestinal cholesterol uptake. (D) Representation of the 26 genes with an FDR < 0.05 extracted from a list of 106 genes from the GSEA pathway for "bile acid metabolism". (E) Bile acid quantification by LC-MS/MS in the plasma and (F) the liver. Data represent the mean ± SEM. n = 4 mice/group. Statistical differences were evaluated using two-tailed student's *t* test (*P* < 0.05). FDR, false discovery rate; GSEA, Gene Set Enrichment Analysis; LC-LDLR, low-density lipoprotein receptor; MS/MS, liquid chromatography-tandem mass spectrometry.

The upregulation of pathways in lipid metabolism (Fig. 3H), together with the reduction in the % of liver/body weight in finasteride-fed mice (supplemental Table S1), prompted us to quantify the hepatic lipid content. Total lipid content assessed by the Folch method revealed a reduction in hepatic lipids in finasteride-fed mice (Fig. 4A). We did not observe differences in hepatic triglyceride secretion or intestinal cholesterol absorption (Fig. 4B, C).

However, our RNA sequencing analysis revealed "bile acid metabolism" as the most upregulated pathway in response to finasteride (Fig. 3H). This pathway contains 106 genes, among which 26 appeared differentially regulated with an FDR < 0.05 (Fig. 4D). Among these 26 genes, the transcription factor *Nr0b2*, also known as small heterodimer partner (SHP), appeared as the most upregulated gene (Fig. 4D). SHP binds and inhibits the transactivation of hormone receptors such as the *Nr1i2* and *Nr1h4*, known as pregnane X receptor (PXR) and farnesoid X receptor (FXR), respectively (55). PXR and FXR dimerize in the nucleus with the retinoid X receptors (*Rxrs*) to regulate gene expression by binding to the promoter of target genes, including those regulating bile acid synthesis. *Nr1i2* and *Rxra* appeared downregulated in response to finasteride, which, together with the upregulation of *Nr0b2*, indicates that finasteride could decrease bile acid synthesis (Fig. 4D).

These results prompted us to quantify the net effect of finasteride on bile acid levels by LC-MS/MS in the plasma and the liver samples. Our data show a trend toward decreasing the total, unconjugated, and taurine/glycine-conjugated bile acids in the plasma and the liver of finasteride-fed mice (Fig. 4E, F). As previously reported, our bile acid quantifications revealed lower glycine–conjugated bile acids compared to taurine conjugation in plasma and liver (56). Bile acid quantifications by colorimetric assay revealed comparable results; mice fed finasteride showed a reduction in total bile acids in the plasma but failed to show significant differences in hepatic bile acids in comparison to littermates fed without finasteride. Analysis of total fecal bile acid content also revealed a trend toward a reduction in finasteride-fed mice (*P* = 0.055) (supplemental Fig. S2).

### Human study—finasteride treatment is associated with a reduction in total plasma cholesterol and LDL-C in the NHANES dataset

**Fig. 5:**
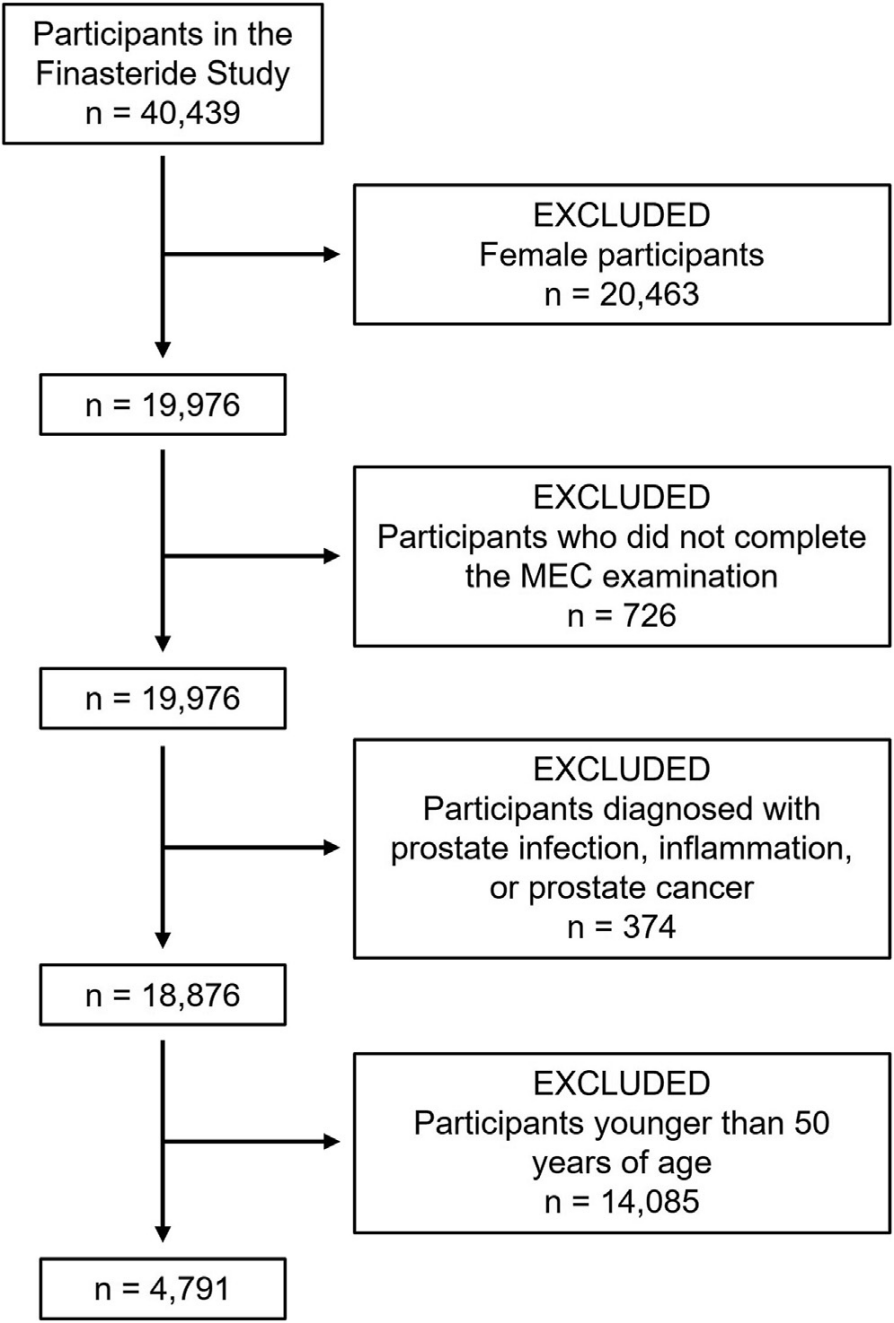
Exclusion flow chart of the study protocol. MEC; mobile examination center.

Our findings in *Ldlr*^*−/−*^ mice indicate that finasteride supplementation reduces triglycerides and total plasma cholesterol without affecting HDL-C levels (Fig. 1C–E). Hence, we examined whether the use of finasteride in humans is associated with changes in the plasma lipid profile using retrospective data obtained from the NHANES database. From a total of 40,439 individuals, 4,791 participants met our inclusion criteria, from which 4,636 that did not take finasteride were compared to 155 participants that reported taking finasteride (Fig. 5). Participant distribution by race/ethnicity and body mass index is summarized in supplemental Table S5. Tables 1 and 2 show the differences in total plasma cholesterol and LDL-C concentrations, respectively, by finasteride use. For the interaction variables assessed, subjects reporting the use of finasteride consistently presented lower levels of cholesterol than those not taking the drug (Table 1). Similarly, LDL-C levels were lower in finasteride users except for those that were neither diagnosed with high cholesterol nor were taking cholesterol-lowering agents (*P* = 0.074, Table 2).

**Table 1:** Total cholesterol analysis of male patients treated with and without Finasteride: NHANES 2009–2016^a^

Total Cholesterol levels in subjects taking finasteride: NHANES 2009-2016						
Interaction variable		No finasteride	Finasteride	*P*	Test for interaction	
		Mean ± SE	Mean ± SE		F	*P*
Alcohol > four or five drinks per day	Yes	174 ± 3	138 ± 8	0.0002	2.78	0.1006
	No	174 ± 3	152 ± 5	0.0001		
Diagnosed with cardiovascular disease	Yes	170 ± 4	124 ± 9	<0.0001	10.6	0.0018
	No	178 ± 3	166 ± 5	0.0253		
Taking drugs that affect glucose metabolism	Yes	171 ± 3	155 ± 5	0.0105	6.12	0.0161
	No	178 ± 4	135 ± 9	<0.0001		

a Statistical differences were considered significant when *P* < 0.05 and *P* < 0.15 for differences in means between No finasteride and Finasteride groups and the Test for interaction, respectively. Cholesterol units: mg/dl.

**Table 2:** LDL cholesterol (LDL-C) analysis of male patients treated with and without Finasteride: NHANES 2009–2016^a^

LDL cholesterol levels in subjects taking finasteride: NHANES 2009-2016						
Interaction variable		No finasteride	Finasteride	p	Test for interaction	
		Mean ± SE	Mean ± SE		F	p
Diagnosed high blood cholesterol (Diagnosed) and/or taking medications for blood cholesterol (Meds)	Diagnosed + Meds	103 ± 6	83 ± 9	0.018	2.3	0.086
	Diagnosed + No meds	129 ± 8	102 ± 10	0.017		
	Not diagnosed + Meds	90 ± 7	61 ± 11	0.017		
	Not diagnosed + No meds	102 ± 5	91 ± 7	0.074		
Taking drugs that affect glucose metabolism	Yes	102 ± 5	87 ± 5	0.008	3.58	0.063
	No	111 ± 6	81 ± 8	0.0002		

a Statistical differences were considered significant when *P* < 0.05 and *P* < 0.15 for differences in means between No finasteride and Finasteride groups and the Test for interaction, respectively. LDL-C units: mg/dl.

We also evaluated the association between finasteride treatment and HDL-C levels (supplemental Table S6). Finasteride treatment was not associated with alterations in HDL-C levels when compared using common interaction variables. supplemental Table S7 shows the results obtained for triglyceride concentration in our dataset. The only significant correlation between finasteride intake and a reduction in triglyceride levels was observed in those subjects diagnosed with high blood cholesterol and currently taking cholesterol-lowering medications (*P* = 0.014). The rest of the interaction variables failed to correlate significantly with alterations in triglyceride levels.

### Human study—associations between finasteride treatment and blood glucose, glycohemoglobin levels in the NHANES dataset

supplemental Table S8 highlights the blood glucose concentrations in the NHANES dataset. From the different interaction variables, finasteride consumption was associated with a reduction in blood glucose in those subjects diagnosed with liver condition (*P* = 0.0418), diagnosed with diabetes, and currently taking antidiabetic medications (*P* = 0.0033), and diagnosed with high blood cholesterol but not currently taking cholesterol-lowering medications (*P* = 0.0101). Our statistical model did not report associations between finasteride intake and the remaining interaction variables. Glycohemoglobin levels in subjects that reported the use of finasteride were lower than those not taking the drug in all the interaction variables but two: nonsmokers and underweight subjects (supplemental Table S9).

Findings regarding glucose metabolism prompted us to examine the effect of finasteride in glucose metabolism in our mouse study. Finasteride failed to alter fasting glucose levels or glucose tolerance tests in *Ldlr*^*−/−*^ mice fed finasteride at any of the doses administered in our study (supplemental Fig. S3).

## Discussion

Upon its accumulation in tissues such as the prostate and the hair follicle, finasteride inhibits the activity of SRD5A2 to block the conversion of circulating testosterone to its active metabolite 5α–DHT. As a result, finasteride mitigates the harmful action of 5α–DHT in these tissues without causing significant side effects. Indeed, over 70 clinical trials demonstrate the safety of finasteride in people, making this drug one of the most prescribed medications in the United States (41, 57, 58, 59, 60, 61). In 1997, finasteride received its FDA approval at an authorized dose as Propecia (1 mg dose) to prevent and treat hair loss, and later in 2003, with the name of Proscar (5 mg dose) for benign prostate hyperplasia (FDA.gov). The use of finasteride has been recently expanded to new populations such as hirsute women and transgender individuals, where finasteride aids in the treatment of hair loss in both trans men and women (62, 63, 64, 65, 66, 67). The length of finasteride treatment can span decades, highlighting the importance of understanding the long-term effects of finasteride per se, as well as on testosterone levels, in slow-progressive diseases such as CVD.

Association studies between testosterone and CVD risk suggest that low levels of endogenous testosterone in men are associated with greater odds of CVD outcomes. However, the effect of exogenous testosterone on CVD risk and atherosclerosis remains elusive: On one hand, the Cardiovascular Testosterone Trial failed to observe alterations in the intima-media thickness of older (+60) cis men with low or low-normal serum testosterone, while a second study with comparable demographics observed an increase in plaque volume (6, 68). On the other hand, a study with trans men exposed to gender-affirming therapy suggest that testosterone could promote shear stress on the endothelial wall vessel (69). Further studies will be necessary to understand whether testosterone or its derivative 5α–DHT contribute to the greater CVD risk observed in the transgender population (70, 71), a demographic increasingly prescribed finasteride to treat androgenic alopecia (62, 63, 64, 67).

While the long-term effect of finasteride on CVD outcomes has not been examined, a few clinical studies have explored the role of finasteride in surrogate indicators of CVD risk, such as plasma lipid levels. These studies led to inconsistent outcomes, probably due to confounding factors such as the treatment duration, participants' age, and study size (72, 73, 74, 75, 76, 77). In preclinical settings, only one study has tested the effect of finasteride supplementation in the development of atherosclerosis. Liu *et al*., showed that finasteride ameliorates plasma lipid profile and reduces atherosclerotic lesion size in apoE-deficient mice. These authors administered 1.5 mg of finasteride/30 g of body weight, which is a comparable dose to our 1000 mg of finasteride/kg of diet, equivalent to approximately 2.5 mg finasteride/mouse. However, the authors failed to examine the association between these observations (19). Here, we show that dietary supplementation with 1000 mg of finasteride/kg diet, a comparable dose utilized by Liu et al., also improves plasma lipid profile, and delays the development of atherosclerosis at two separate anatomical points of the aorta in *Ldlr*^*−/−*^ mice.

An essential outcome of this report is the parallelism between our preclinical and population-based studies regarding plasma cholesterol levels, one of the main drivers of atherosclerotic CVD in people (78). We show that finasteride intake was associated with a reduction in total cholesterol and LDL-C levels in the NHANES dataset, as it occurred in total plasma cholesterol in our mouse study (Tables 1 and 2, and Fig. 1C). In both cases, HDL-C remained unaltered regardless of finasteride supplementation (supplemental Table S6 and Fig. 1D). The parallelism between both studies, however, is not complete. We report a significant reduction in plasma triglyceride in mice fed 1000 mg/kg of finasteride. Still, most of the associations in the NHANES dataset failed to reach statistical significance for this parameter, despite showing a trend toward decreasing in participants taking finasteride (Fig. 1E and supplemental Table S7). These discrepancies could be attributed to a greater variability in fasting triglyceride levels than those observed in plasma cholesterol, which is estimated to reach up to 28% in comparison to a 6%–9%, respectively (79). Regarding glucose metabolism, our preclinical study failed to mimic the positive outcomes on glucose and glycohemoglobin we observed in the NHANES dataset in those participants taking finasteride. These differences could be attributed to interspecific responses to finasteride or the dramatic differences between plasma lipid levels between humans and *Ldlr*^*−/−*^ mice fed Western diet. Studies in WT mice to examine whether finasteride improves glucose tolerance would be useful to explore the discrepancies in our results.

Considering the results in our dose-response study in *Ldlr*^*−/−*^ mice, where only the greatest dose of finasteride resulted in alterations in plasma lipid levels, it is plausible to assume that a high dose of finasteride might be necessary to achieve its beneficial effects on plasma lipid profile in people. The prevalence of prostate hyperplasia increases with age, and because our inclusion criteria focused on men above 50 years of age, we could assume that finasteride was prescribed at the dose of 5 mg/day (61). We could predict that those subjects taking 1 mg finasteride/day, which is prescribed for hair loss, would not experience an improvement in plasma lipid profile. Future studies will be necessary to establish these assumptions. Taken together, the parallelisms between our preclinical and population-based studies suggest that finasteride supplementation could ameliorate atherosclerosis in people when prescribed at a high dose and, therefore, prevent the development of CVD.

We utilized our preclinical study to tease out the potential mechanism(s) of action by which finasteride decreases atherosclerosis. To this end, we explored the effect of finasteride on circulating monocytes and plaque composition. Finasteride reduced monocytosis without altering the Ly6c^hi/lo^ ratio, an indicator of monocyte inflammatory status in mice (42). These results were accompanied by a net reduction in macrophage content and monocyte recruitment to the lesion (Fig. 2F–K). While we did not explore whether finasteride directly alters monocyte progenitor cells in the bone marrow, alterations in monocyte number could be attributed to a reduction in plasma cholesterol (Fig. 1C) (42, 80). We cannot discard direct effects of finasteride or the variations in sex hormones on endothelial permeability and/or macrophage polarization status. Indeed, androgen signaling is positively associated to endothelial dysfunction and a proinflammatory macrophage phenotype (69, 81, 82, 83). Regardless, the modifications in plaque size were associated with plasma cholesterol levels, suggesting that this parameter drives the development of atherosclerosis in our experimental model (supplemental Fig. S1). Notably, these changes occurred without alterations in body weight, food intake, or liver damage, as reported by our transaminase activity assays and miR-122 plasma levels (supplemental Table S1).

We next switched our attention to the effect of finasteride in the liver as one of the key regulators of plasma lipid profile, one of the main drivers of atherosclerosis development (45). Surprisingly, the effect of finasteride on hepatic metabolism has been understudied, possibly due to the low expression of SRD5A2 in the hepatocyte (84). Our RNA sequencing data, liver weight, and composition suggest that finasteride intake had beneficial effects on hepatic metabolism in our experimental model. GSEA suggested that finasteride decreases hepatic inflammation, in agreement with a downregulation of monocyte/macrophage markers in liver homogenates exposed to finasteride. In line with these results, preclinical studies show that atherosclerosis and liver injury is associated with increased leukocyte infiltration in the liver (85, 86, 87), as it also occurs with CD8+ cells (88). While we did not analyze the macrophage or CD8+ cell content in the liver, our RNA sequencing data suggest that finasteride depletes the relative amount of these cells in the liver (Fig. 3I).

Our RNA sequencing data also reveal an upregulation of lipid pathways, and our Folch analysis shows that finasteride-fed mice presented lower fat content in the liver compared to littermates fed a finasteride-free diet (Figs. 3H and 4A). Regarding the "bile acid metabolism" pathway, our bile acid quantifications using mass spectrometry-based and colorimetric assays suggest that finasteride depletes bile acid production and excretion of these cholesterol metabolites. These changes could be attributed to alterations caused by finasteride or its derivatives in the regulation of FXR:RXR and/or PXR:RXR pathways, or the reduction in plasma cholesterol, which is necessary for bile acid production. While we did not directly explore whether finasteride increases hepatic oxidative phosphorylation or the net contribution of finasteride to "cholesterol homeostasis", we examined the genes differentially regulated with an FDR < 0.05 in our RNA sequencing dataset (supplemental Fig. S4A, B). Several genes in the oxidative phosphorylation pathway indicated an increased mitochondrial activity, which could result in the overproduction of radical oxygen species (ROS) (89). However, after examining classical markers upregulated in response to oxidative stress, we ruled out this possibility, as we observed that finasteride failed to upregulate the expression of ROS-sensitive genes. On the contrary, finasteride downregulated *Hmox1* mRNA expression, which could be interpreted as a reduction of ROS levels in the liver in finasteride-fed mice (90) (supplemental Fig. S4C).

We also explored whether finasteride exposure alters the expression of carboxylesterases, a group of enzymes implicated in phase I xenobiotic catabolism and the hydrolysis of triglycerides, cholesteryl esters, and retinyl esters (91). To date, a total of six human and 20 murine carboxylases have been identified. Our RNA sequencing data show that four of these murine carboxylesterases (*Ces1c*; *Ces1e*; *Ces1g*; and *Ces2a*), all of which are highly expressed in the liver (91), were upregulated by finasteride (supplemental Fig. S5). *Ces1c*, *Ces1e*, and *Ces1g* display retinyl ester hydrolase activity, suggesting that finasteride could modulate hepatic retinoid homeostasis. Global ablation of *Ces1g* in mice results in weight gain, insulin resistance, liver steatosis, and hyperlipidemia through upregulation of de novo lipogenesis and oversecretion of TG-rich lipoproteins (92). Selective restoration of *Ces1g* expression only in the liver significantly reduced hepatic TG-storage accompanied by decreased size of lipid droplets, reduced VLDL-secretion, and improved insulin signaling in the liver, thus hepatic *Cesg1* plays a critical role in limiting hepatic steatosis, VLDL assembly and in augmenting insulin sensitivity (91, 92). Finasteride also upregulated *Ces2a* expression which could contribute to explaining the phenotype we observe in our mice. The role of *Ces2a* in lipid metabolism was recently unveiled by the generation of a mouse model lacking this enzyme (93). *Ces2a*^*−/−*^ mice displayed hepatic steatosis and impaired insulin resistance involving impaired diacylglycerol and lysophosphatidylcholine catabolism (93). Future experiments should address the contribution of carboxylesterases as potential mediators in the role of finasteride on hepatic and systemic lipid metabolism.

Sex hormones have a profound impact on gene expression in tissues including the liver, where they regulate inflammation and lipid metabolism (recently reviewed in (94, 95)). Finasteride treatment resulted in a shift in circulating sex hormones and hepatic sex hormones toward estrogen excess, as reported in the past (18, 50, 61, 72). Our LC-MS data revealed an increase in hepatic estradiol in mice fed finasteride, which could be responsible, at least in part, for the reduction in hepatic lipid levels and antiinflammatory effects in our experimental model (Fig. 3F, G).

There are remarkable differences between the xenobiotic detoxification pathways between rodents and humans, which complicates the establishment of parallelisms regarding drug dosages among different species (96). However, the upregulation of the pathway named "xenobiotic metabolism" in finasteride-exposed mice could be interpreted as hepatic toxicity (97), which would agree with some reports suggesting that finasteride causes liver damage and other alterations (98). While side effects are considered rare, recent findings shed light on possible adverse health outcomes of finasteride in people (99). We failed to observe alterations in circulating markers for liver damage in response to any dose of finasteride compared to finasteride-free diet-fed littermates that, together with the absence of body weight or food intake alterations, allow us to rule out toxic effects in our experimental model (supplemental Table S1).

Our mouse study suggests that the prostate is highly sensitive to finasteride, while the effects of finasteride in the liver require a greater dose. Indeed, *Cyp3a11*, *Ugt1a5*, and SHP mRNA expression in the liver were only affected in those fed 1000 mg of finasteride/kg of diet (supplemental Fig. S6). However, finasteride reduced the prostate size in a dose-response manner, an effect that was evident at our lowest dose (10 mg/kg dose) (Fig. 1A, B). Taken together, our population-based and preclinical studies indicate that finasteride treatment ameliorates plasma lipid profile. Our mouse study also shows that finasteride reduces the burden of atherosclerosis, with an overall positive effect on the liver. Considering that finasteride has been safely prescribed for decades, these results will pave the road toward using finasteride, alone or in combination with other medications, to tackle CVD in people.

### Limitations of the study

Our study describes parallelisms between mice and humans on plasma cholesterol in response to finasteride. However, these results must be interpreted with caution due to, at least in part, interspecific differences on lipoprotein metabolism. For example, mice do not express cholesteryl ester transfer protein, an enzyme that catalyzes the movement of cholesteryl ester between HDL and apoB-containing lipoproteins (100). Additionally, mice produce apoB48 and apoB100-containing chylomicrons and VLDL, while humans produce exclusively apoB48 chylomicrons and apoB100 VLDLs. Murine apoB48 VLDL/LDL particles are secreted and cleared at a greater rate than those containing apoB100, and these particles also lack the apoB binding site for the LDLR (101, 102).

Several limitations in our human retrospective study also deter us from drawing conclusive information. For example, our study does not include plasma testosterone levels in participants, nor we can estimate the adherence to finasteride, dosage (1 vs. 5 mg/day), or duration of the treatment. A relatively low number of participants taking finasteride met our inclusion criteria (n= 155), and therefore, we lacked statistical power to examine whether finasteride interacted differently with drugs aiming to reduce cholesterol synthesis (statins) and those targeting cholesterol intestinal absorption (ezetimibe) (Table 2, supplemental Tables S7 and S8).

A previous report suggests that the mechanism of action of finasteride on atherosclerosis depends on its effects at the level of the microbiota. The authors showed that finasteride reduces trimethylamine N-oxide (TMAO) production in the gut and plasma levels (19). The same authors recently reported that the reduction in TMAO mediates the positive effects of finasteride on nephropathy induced by protein overload in mice (103). In our study, however, we did not quantify TMAO levels in tissues or feces, nor comprehensively examined the effect finasteride at the intestinal level other than studying cholesterol uptake (Fig. 4C).

Regardless of these limitations, our data provide an important steppingstone toward repurposing the use of finasteride for the prevention and treatment of atherosclerotic cardiovascular disease.

## Data Availability

The data described in this article are presented in the figures, tables, or supplemental material. The RNA sequencing data that support the findings of this study are available in GSE255708 (https://www.ncbi.nlm.nih.gov/geo/query/acc.cgi?acc=GSE255708). Additional data are available on request from the corresponding author.

## Supplemental data

This article contains supplemental data.

## Conflict of interest

The authors declare that they have no conflicts of interest with the contents of this article.
