# Supplementary material for: Finasteride delays atherosclerosis progression in mice and is associated with a reduction in plasma cholesterol in men
Source: J Lipid Res. 2024 Jan 23;65(3):100507. doi: 10.1016/j.jlr.2024.100507 (PMC10899056; doi:10.1016/j.jlr.2024.100507)
Supplement: Supplementary Figures [file mmc1.docx]

**Supplementary Figures**


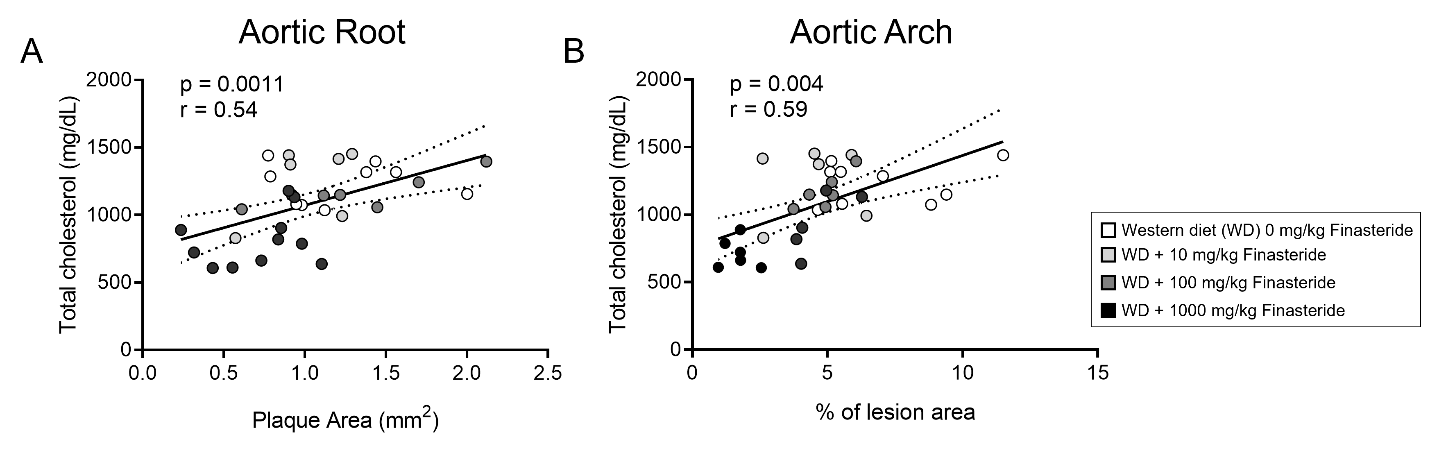


**Supplementary Figure 1.** **Correlation between plaque size and total cholesterol in *Ldlr^-/-^* mice.** Four-week old male *Ldlr^-/-^* mice were fed Western diet with increasing doses of finasteride for 12 weeks before tissue and blood harvesting. **(A)** Lesion area at the level of the aortic root, and **(B)** aortic arch were plotted with total cholesterol levels at the moment of the sacrifice.

**
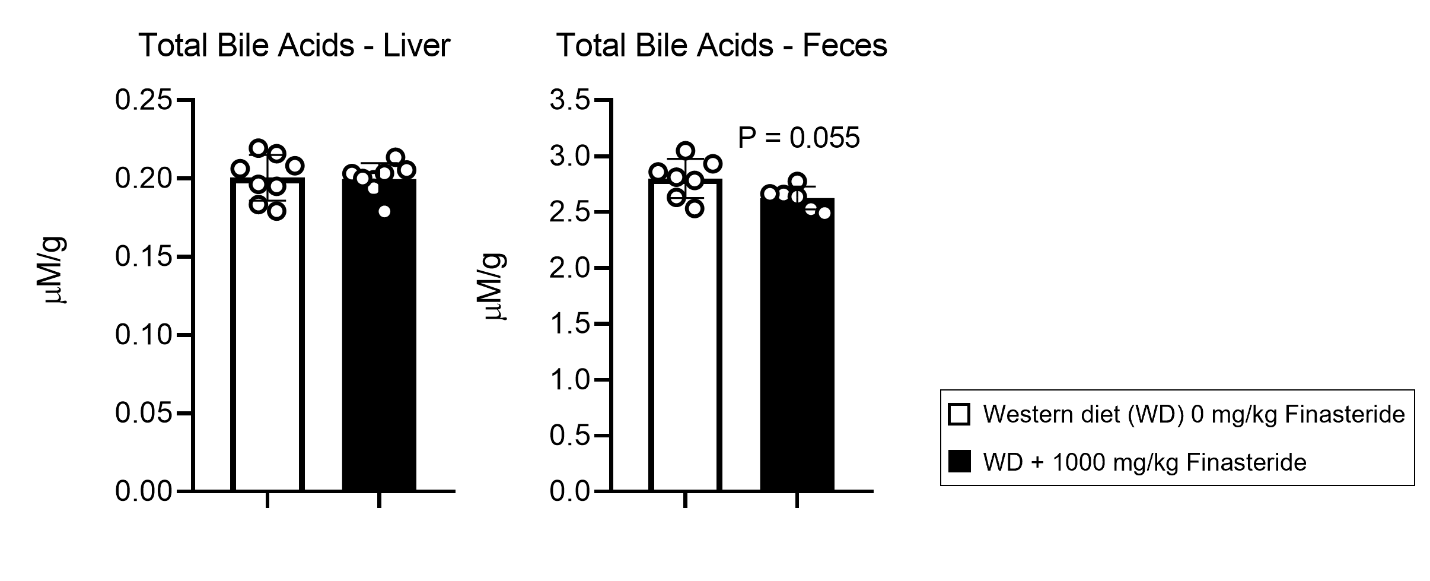
**

**Supplementary Figure 2. Total bile acid levels.** Four-week-old male *Ldlr^-/-^* mice were fed either Western diet with 1000 mg finasteride/kg or the same diet without finasteride for 12 weeks. Total bile acid levels were measured by a colorimetric assay using (Diazyme, Total Bile Acid Assay) in the plasma, liver, and feces. Data represent the mean ± SEM. Statistical differences were evaluated using two-tailed student’s t-test (p < 0.05). n = 5 to 8 mice/group. *** p < 0.005.


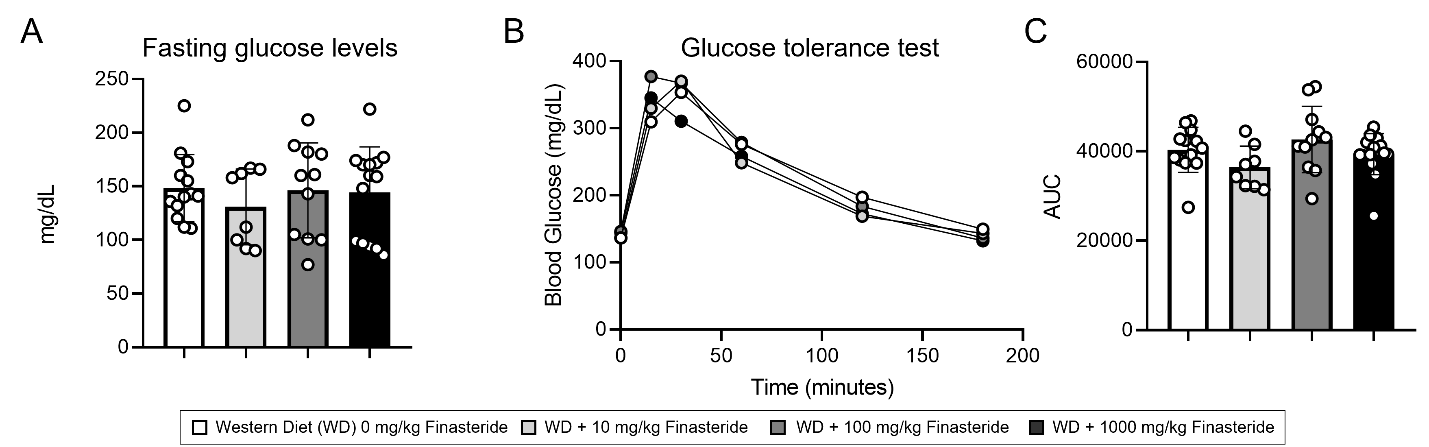


**Supplementary Figure 3.** **Fasting glucose and glucose tolerance test assay.** Four week-old male *Ldlr^-/-^* mice were fed Western diet with increasing doses of finasteride for 11 weeks before performing a glucose tolerance test (see methods for more details). **(A)** Fasting glucose levels, and **(B)** Glucose tolerance tests were performed under fasting conditions. **(C)** Area under the curve (AUC) calculated for each animal. Data represent the mean ± SEM. Statistical differences were evaluated using one-way ANOVA (p < 0.05).


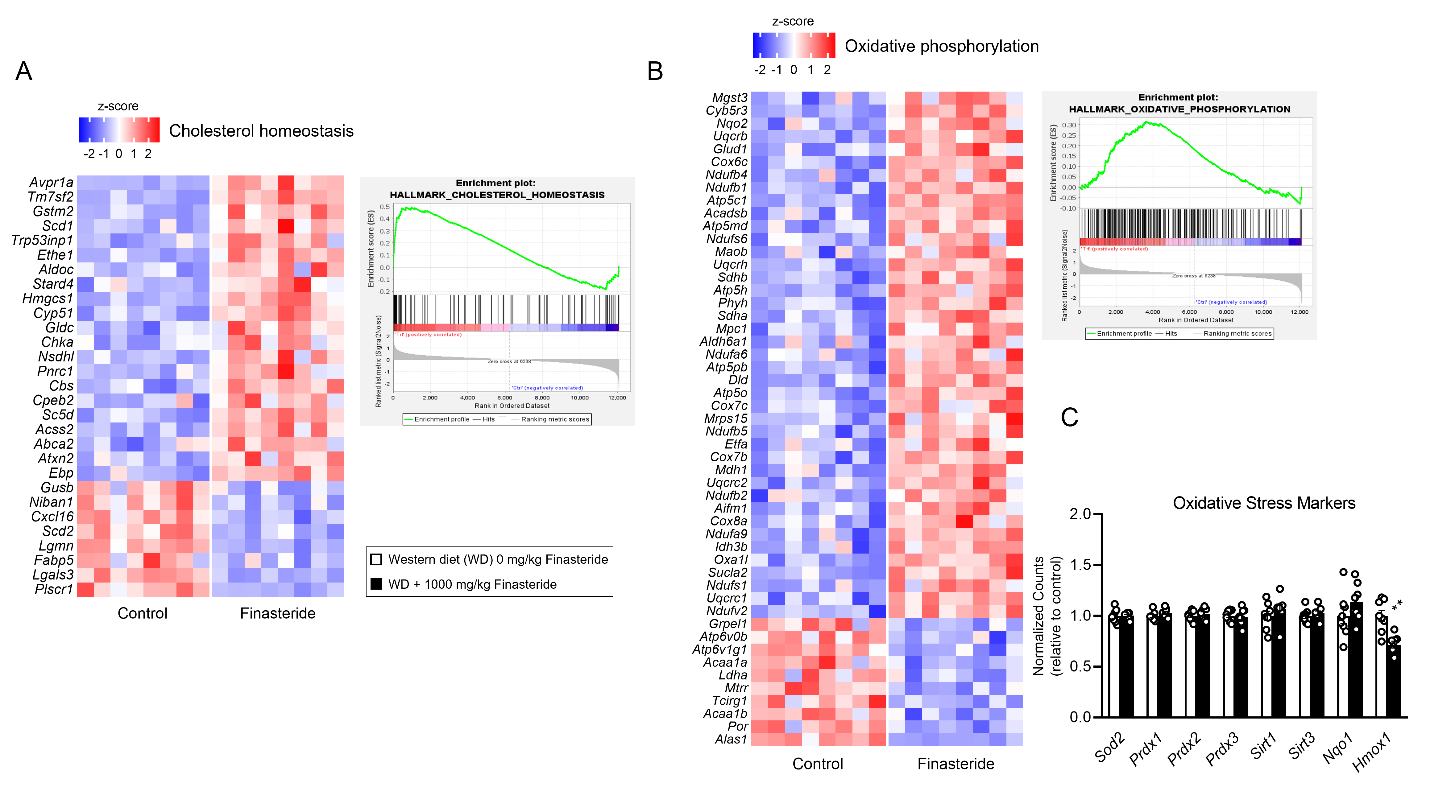


**Supplementary Figure 4.** Four-week-old male *Ldlr^-/-^* mice were fed either Western diet with 1000 mg finasteride/kg or the same diet without finasteride for 12 weeks. Total liver mRNA was extracted and sequenced. **(A)** Representation of the 29 genes with an FDR < 0.05 extracted from a list of 72 genes from the GSEA pathway for "Cholesterol homeostasis". **(B)** Representation of the 51 genes with an FDR < 0.05 extracted from a list of 199 genes from the GSEA pathway for "Oxidative phosphorylation" **(C)** Expression of genes responsive to reactive oxygen species extracted from the RNA seq dataset. Data extracted from the RNA seq were evaluated using FDR. ** q < 0.01 considering *Ldlr^-/-^* mice fed Western diet without finasteride as reference group.

**
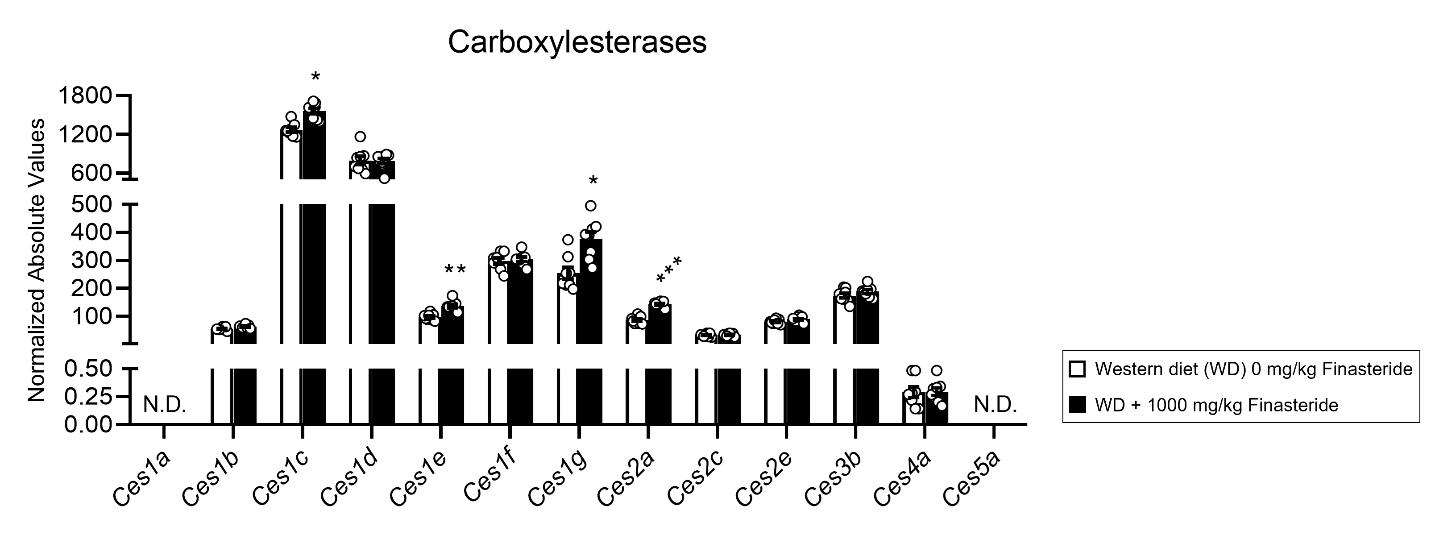
**

**Supplementary Figure 5** Four-week-old male *Ldlr^-/-^* mice were fed either Western diet with 1000 mg finasteride/kg or the same diet without finasteride for 12 weeks. Total liver mRNA was extracted and sequenced. Data represent the expression of murine carboxylesterases. Data represent the mean ± SEM. Statistical differences were evaluated using FDR. * q < 0.05, ** q < 0.01, *** q < 0.005. N.D: Not detected.


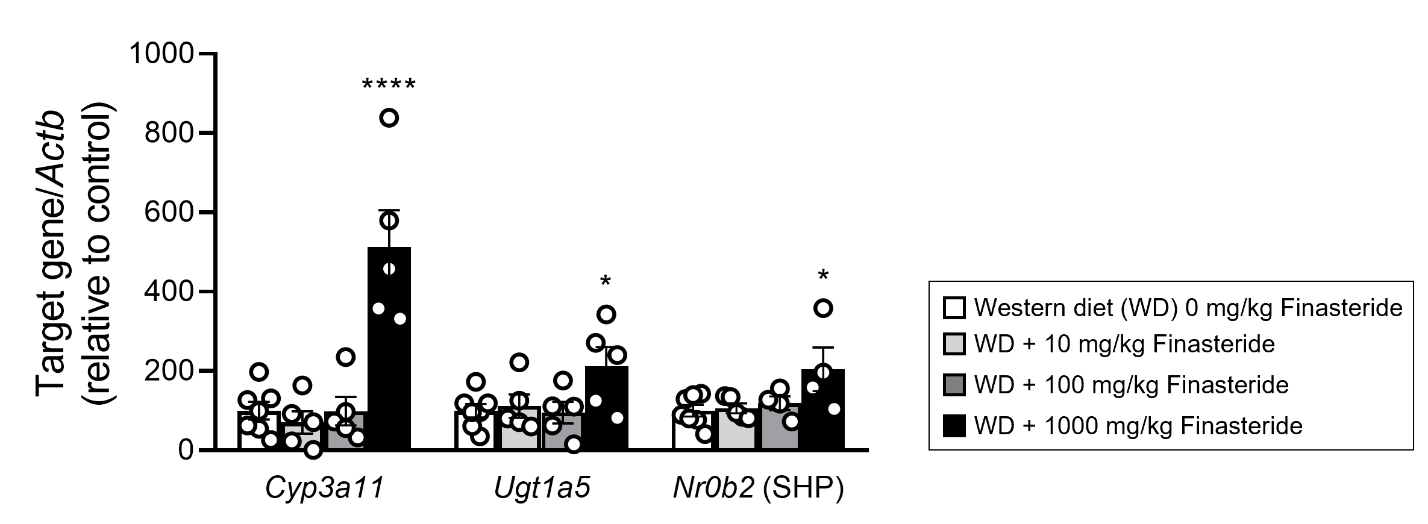


**Supplementary Figure 6.** Four-week-old male *Ldlr^-/-^* mice were fed Western diet with increasing doses of finasteride for 12 weeks (see methods for more details). RT-PCR results for total liver homogenates. Data represent the mean ± SEM. Statistical differences were evaluated using one-way ANOVA (p < 0.05). n = 5 to 6 mice/group. * p < 0.05, **** p < 0.001 considering *Ldlr^-/-^* mice fed Western diet without finasteride as reference group.
