# Supplementary material for: Finasteride delays atherosclerosis progression in mice and is associated with a reduction in plasma cholesterol in men
Source: J Lipid Res. 2024 Jan 23;65(3):100507. doi: 10.1016/j.jlr.2024.100507 (PMC10899056; doi:10.1016/j.jlr.2024.100507)
Supplement: Supplementary Tables [file mmc2.docx]

**Supplementary Tables**

**Supplementary Table 1.** **Food intake, body composition, and liver toxicity in *Ldlr^-/-^* mice fed finasteride.** ALT, alanine transaminase; AST, aspartate aminotransferase. Data represent the mean ± SEM. Statistical differences were evaluated using one-way ANOVA (p < 0.05). n = 8 to 15 mice/group. * p < 0.05 considering *Ldlr^-/-^* mice fed Western diet without finasteride as a reference group.


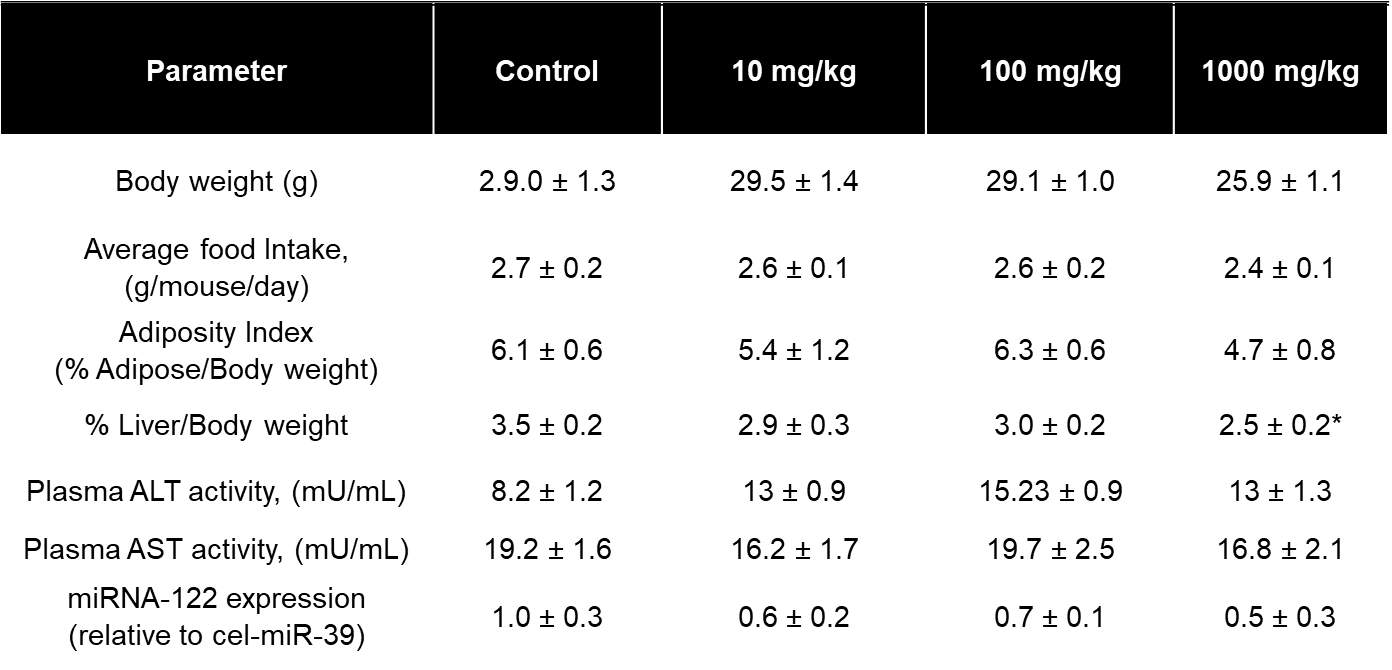


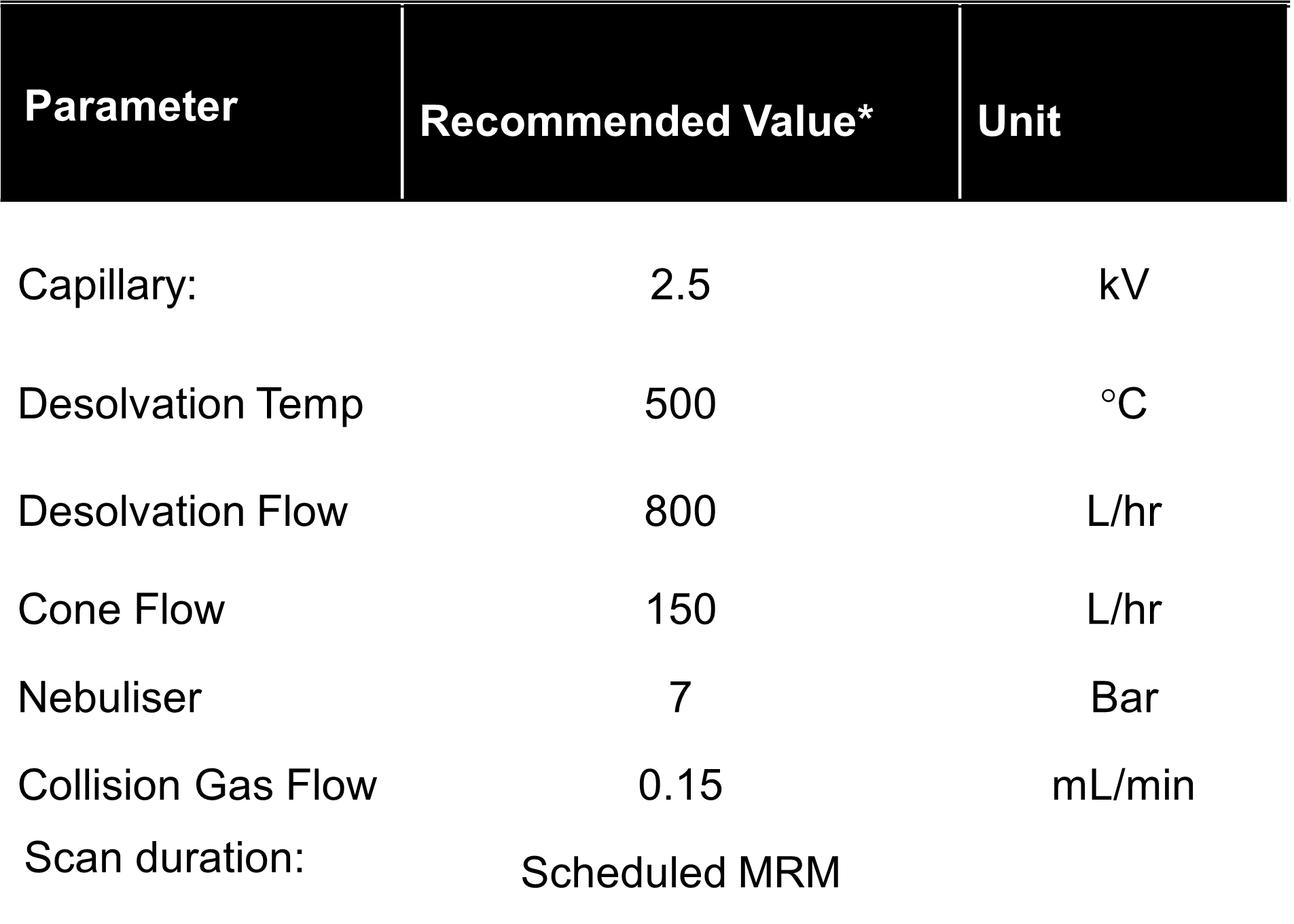
**Supplementary Table 2. LC-MS/MS Program for Bile Acid Analysis.**

**Supplementary Table 3.** **LC-MS/MS Acquisition Method Parameters for Bile Acid Quantification^1^.**


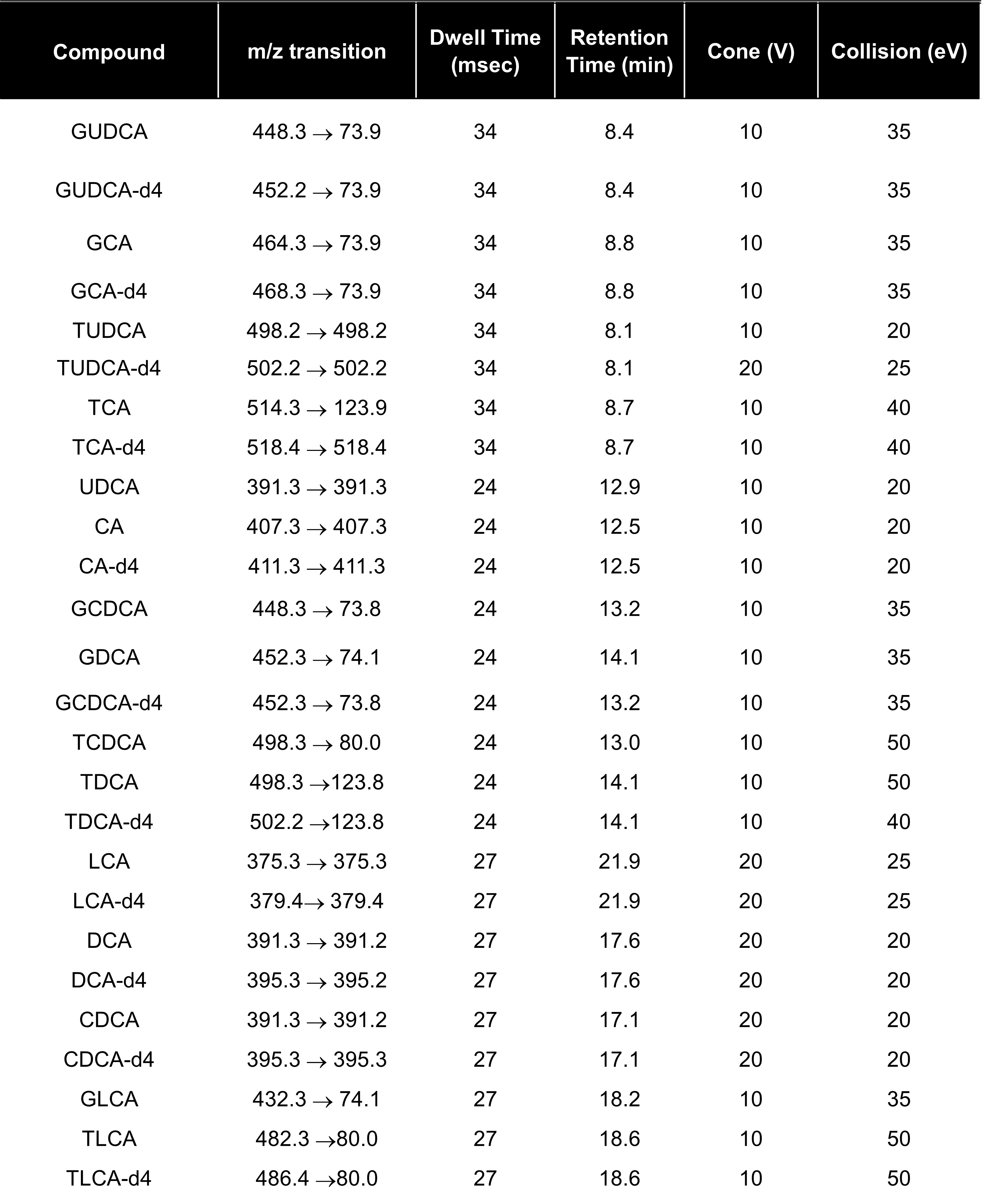


**^1^Abbreviations:** CA: cholic acid (LMID: LMST04010001); CA-d_4_: cholic acid (2,2,4,4-d_4_); CDCA: chenodeoxycholic acid (LMID: LMST04010032); DCA: deoxycholic acid (LMID: LMST04010040); GCA: glycocholic acid (LMID: LMST05030001); GCDCA: glycochenodeoxycholic acid (LMID: LMST05030008); GCDCA-d_4_: glycochenodeoxycholic acid (2,2,4,4-d_4_); GDCA: glycodeoxycholic acid (LMID: LMST05030006); GLCA: glycolithocholic acid (LMID: LMST05030009); GUDCA: glycoursodeoxycholic acid (LMID: LMST05030016); LCA: lithocholic acid (LMID: LMST04010003); TBA: total bile acids; TCA: taurocholic acid (LMID: LMST05040001); TCDCA: taurochenodeoxycholic acid (LMID: LMST05040005); TDCA: taurodeoxycholic acid (LMID: LMST05040013); TLCA: taurolithocholic acid (LMID: LMST05040003); UDCA: ursodeoxycholic acid (LMID: LMST04010033)

**Supplementary Table 4.** LC-MS/MS Acquisition Method Parameters for Steroid Quantification.

**Supplementary Table 5.** Descriptive statistics NHANES 2009-2016.


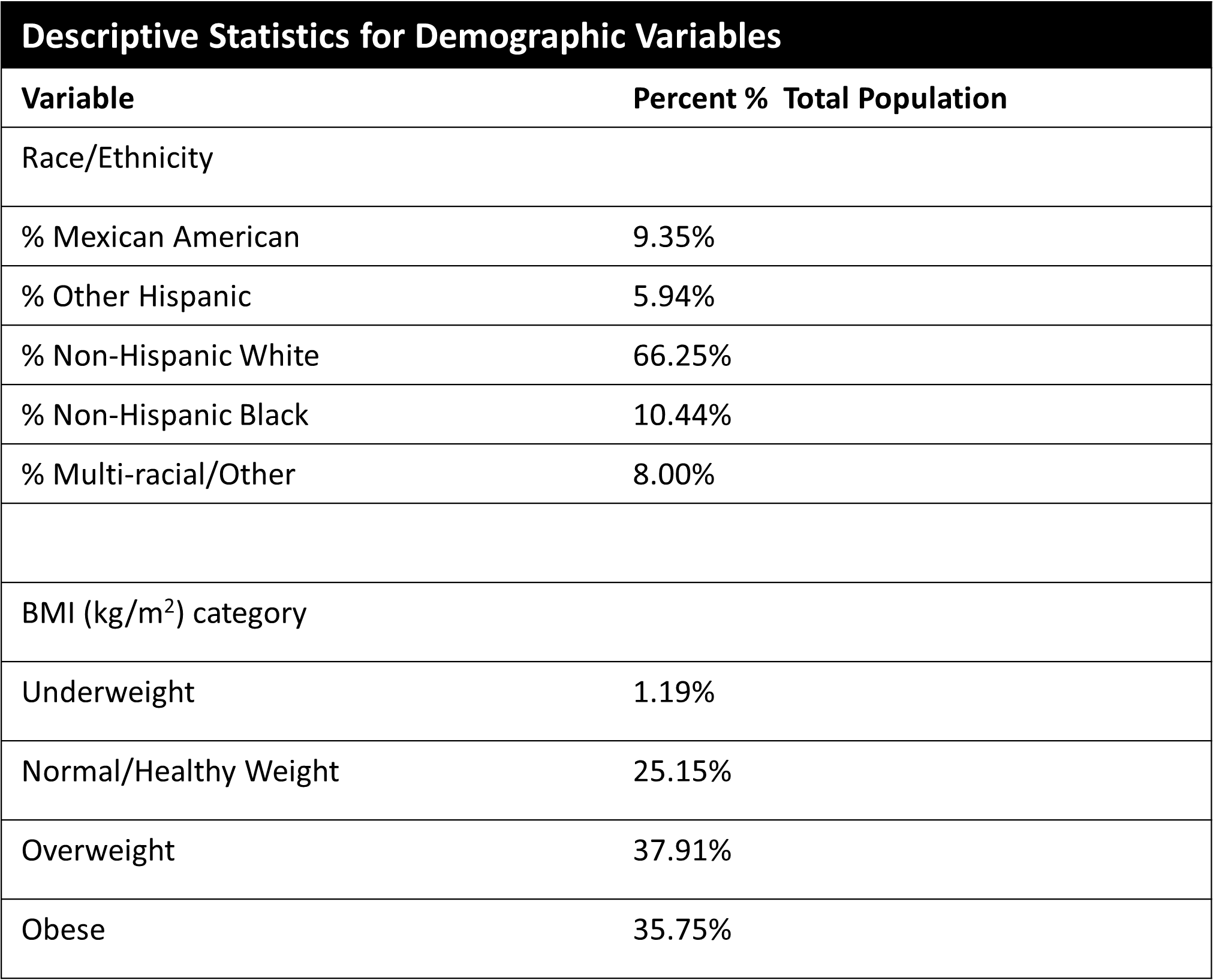


**Supplementary Table 6.** HDL analysis of male patients treated with and without Finasteride: NHANES 2009-2016^1^


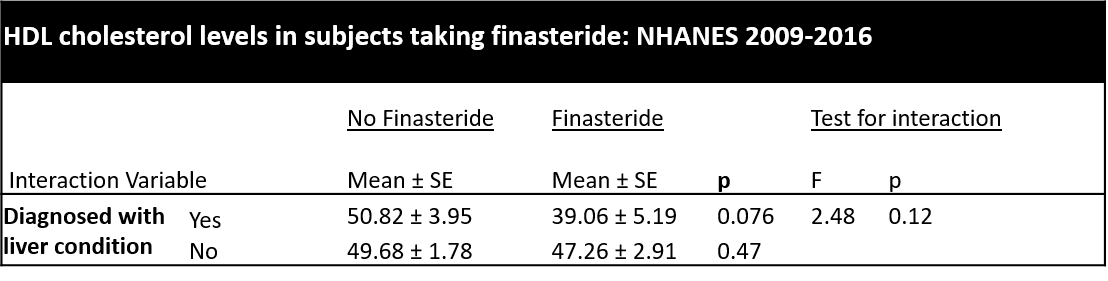


^1^Footnote: Statistical differences were considered significant when p < 0.05 and p < 0.15 for differences in means between No finasteride and Finasteride groups and the Test for interaction, respectively. HDL-C units: mg/dL.

**Supplementary Table 7.** Triglyceride analysis of male patients treated with and without Finasteride: NHANES 2009-2016


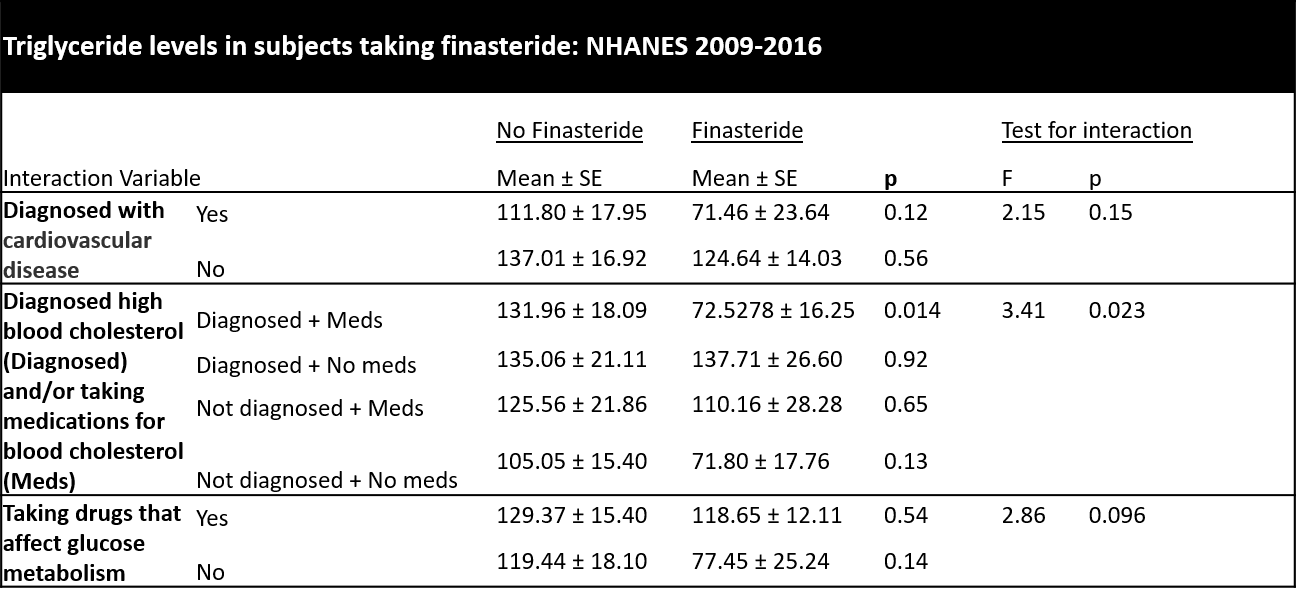


^1^Footnote: Statistical differences were considered significant when p < 0.05 and p < 0.15 for differences in means between No finasteride and Finasteride groups and the Test for interaction, respectively. Triglyceride units: mg/dL.

**Supplementary Table 8.** Association between finasteride treatment and glucose concentration: NHANES 2009-2016^1^


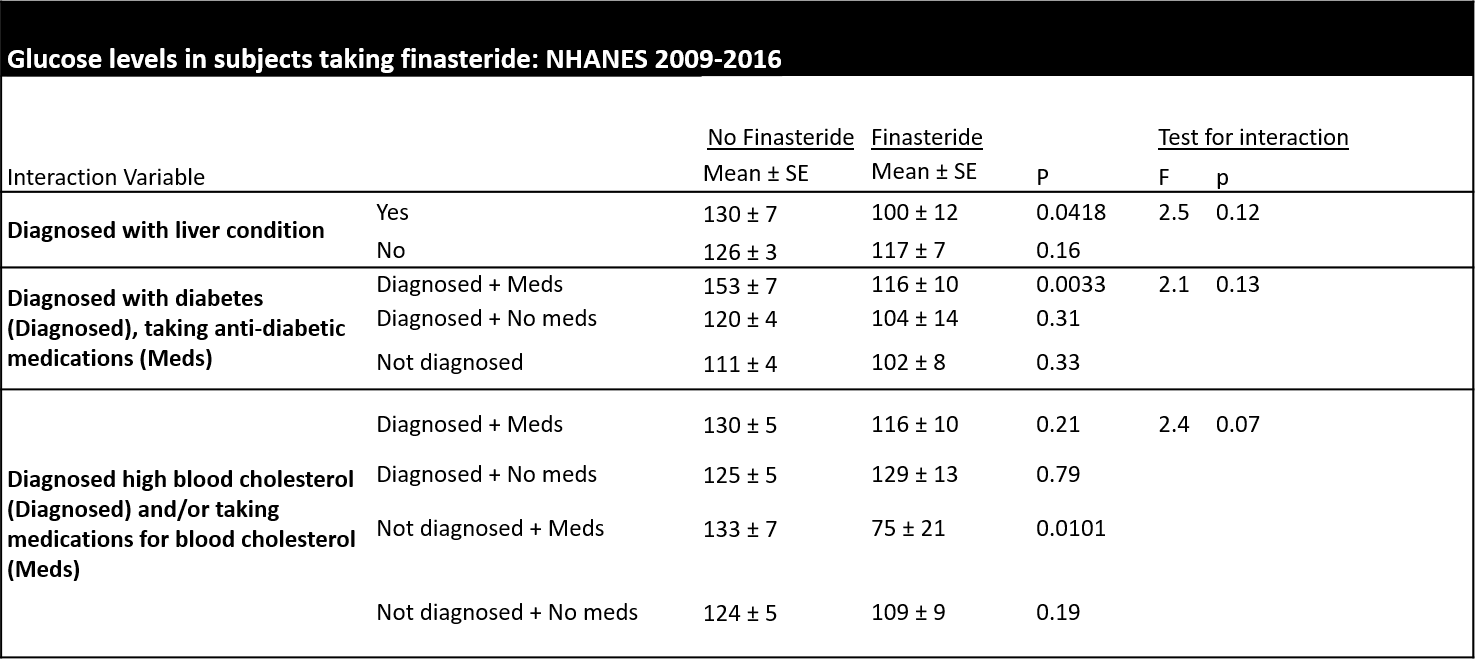


^1^Footnote: Statistical differences were considered significant when p < 0.05 and p < 0.15 for differences in means between No finasteride and Finasteride groups and the Test for interaction, respectively. Glucose units: mg/dL.

**Supplementary Table 9.** Association between finasteride treatment and glycohemogoblin concentration: NHANES 2009-2016^1^


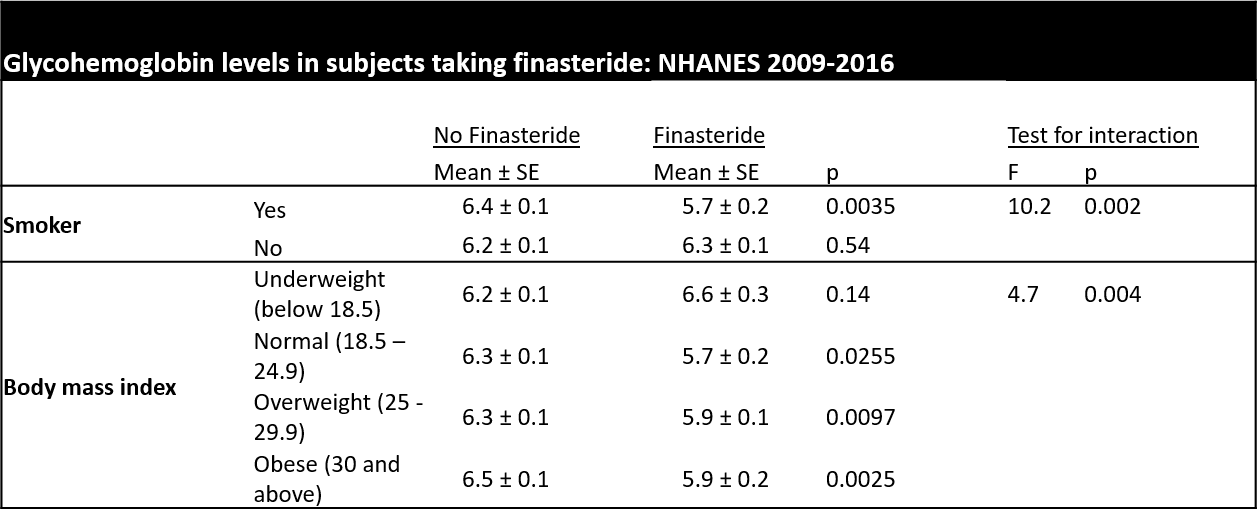


^1^Footnote: Statistical differences were considered significant when p < 0.05 and p < 0.15 for differences in means between No finasteride and Finasteride groups and the Test for interaction, respectively. Glycohemoglobin units: %.
